# A Closed‐Loop‐Capable Neural Interface Platform for Deep Brain Modulation via Integrated Non‐Viral Gene Delivery, NIR Optogenetics, and Electrophysiological Recording

**DOI:** 10.1002/advs.202515060

**Published:** 2025-11-19

**Authors:** Chao‐Yi Chu, Zih‐Huei Chen, Chun‐Wei Liang, Pu‐Wei Wu, Wei‐Qing Guo, Bo‐Wei Chen, Chih‐Chia Huang, Ssu‐Ju Li, Ching‐Wen Chang, Yao‐Wen Liang, Shun‐An Kan, Yu‐Chun Lo, Wei‐Chen Huang, You‐Yin Chen, San‐Yuan Chen

**Affiliations:** ^1^ Department of Materials Science and Engineering National Yang Ming Chiao Tung University No. 1001, Daxue Rd. Hsinchu 300093 Taiwan, ROC; ^2^ Department of Biomedical Engineering National Yang Ming Chiao Tung University No.155, Sec.2, Linong St. Taipei 112304 Taiwan, ROC; ^3^ Department of Photonics National Cheng Kung University No.1, University Rd. Tainan 701401 Taiwan, ROC; ^4^ Center of Applied Nanomedicine National Cheng Kung University No.1, University Rd. Tainan 701401 Taiwan, ROC; ^5^ Department of Education Taipei Veterans General Hospital No.201, Sec. 2, Shipai Rd. Taipei 11217 Taiwan, ROC; ^6^ Ph.D. Program in Medical Neuroscience College of Medical Science and Technology Taipei Medical University 12F., Education & Research Building, Shuang‐Ho Campus, No. 301, Yuantong Rd. New Taipei 23564 Taiwan, ROC; ^7^ Department of Electronics and Electrical Engineering National Yang Ming Chiao Tung University No. 1001, Daxue Rd. Hsinchu 300093 Taiwan, ROC; ^8^ Graduate Institute of Biomedical Science China Medical University No. 91, Xueshi Rd. Taichung 404328 Taiwan, ROC; ^9^ School of Dentistry, College of Dental Medicine Kaohsiung Medical University No. 100, Shiquan 1st Rd Kaohsiung City 807378 Taiwan

**Keywords:** electroporation‐mediated gene delivery, gold inverse opal (AuIO), neural interface, optogenetics, upconversion nanoparticles (UCNPs)

## Abstract

Closed‐loop neuromodulation requires precise, stable, and cell‐specific control of neural circuits with minimal invasiveness. However, conventional optogenetic systems are hindered by invasive optical fibers, viral‐based gene delivery, and disjointed hardware modules. Here, a multifunctional neural interface integrating non‐viral delivery of AAV‐derived Channelrhodopsin‐2 (ChR2) gene plasmid, fiberless intracranial optogenetic stimulation via externally delivered near‐infrared (NIR) excitation, and electrophysiological recording into a single implantable device is reported. The core of this interface is a 3D gold inverse opal (AuIO) microelectrode that provides high surface area, promoting both electroporation‐mediated gene transfection and neural signal acquisition. ChR2‐expressing plasmid DNA is complexed with polyethyleneimine‐neurotensin (NT‐PEI) as non‐viral gene vectors that are immobilized onto designated electrode sites for neuron‐targeted localized gene expression. Upconversion nanoparticles (UCNPs) embedded in a gelatin methacryloyl (GelMA) matrix are precisely integrated onto the microscale AuIO surface via aerosol jet printing, enabling localized surface plasmon resonance (LSPR)‐enhanced NIR‐to‐blue light conversion for remote optogenetic activation. In vivo implantation into the hippocampal dentate gyrus (DG) demonstrates successful opsin expression and real‐time light‐evoked neural activity via single surgical step. This all‐in‐one platform provides a fiber‐free, biocompatible neural interface capable of stable in vivo operation for deep‐brain optogenetic engineering, paving the way for precision closed‐loop neuromodulation.

## Graphical abstract

A multifunctional, 3D porous neural interface combines non‐viral gene delivery and NIR optogenetics to enable minimally invasive, closed‐loop modulation of deep‐brain circuits.

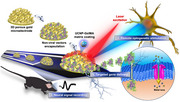

## 1 Introduction

Closed‐loop neuromodulation has emerged as a transformative paradigm in both neuroscience research and clinical neuroengineering, enabling real‐time monitoring and adaptive intervention of neural circuit dynamics.^[^ ^1^, ^2^ ^]^ By continuously adjusting stimulation parameters in response to ongoing neural activity, closed‐loop systems provide temporally precise and functionally specific modulation, thereby addressing the limitations of conventional open‐loop stimulation approaches.^[^ ^3^ ^]^ This strategy has shown significant therapeutic potential in managing a range of neurological and psychiatric disorders, including epilepsy,^[^ ^4^ ^]^ major depressive disorder,^[^ ^5^, ^6^ ^]^ and Parkinson's disease.^[^ ^7^ ^]^ Among currently adopted clinical technologies, deep brain stimulation (DBS) represents a major milestone; however, its efficacy is constrained by the non‐selective nature of electrical stimulation. The electrical fields generated by DBS electrodes affect all neural elements within the target area including excitatory and inhibitory neurons, as well as passing axonal fibers without the capacity to target specific cell types.^[^ ^8^ ^]^ Moreover, its therapeutic mechanism largely relies on sustained high‐frequency depolarization to suppress pathological oscillatory activity,^[^ ^9^, ^10^ ^]^ ultimately aiming to restore functional neural network dynamics.^[^ ^11^. In contrast, optogenetic stimulation offers unparalleled cell‐type specificity^[^ ^12^ ^]^ and millisecond temporal resolution^[^ ^13^, ^14^ ^]^ by genetically introducing light‐gated ion channels (e.g., Channelrhodopsin) into defined neuronal populations. This enables selective activation or inhibition of neurons in response to light exposure,^[^ ^14^ ^]^ offering a powerful tool for elucidating causal relationships within neural circuits and for the development of targeted therapeutic strategies.^[^ ^15^ ^]^ Nonetheless, despite recent progress, the implementation of fully functional, closed‐loop neuromodulation systems remains constrained by several engineering and technological challenges, including real‐time signal processing, miniaturization, wireless operation, and long‐term reliability in vivo.^[^ ^16^ ^]^

Despite the transformative potential of optogenetics, its clinical translation, particularly for chronic applications in deep brain regions, remains hindered by significant technological limitations. Most current systems rely on tethered optical fibers or rigid optoelectronic implants to deliver light, increasing the risks of surgical trauma, chronic neuroinflammation, and spatial misalignment over time.^[^ ^17^ ^]^ While MEMS‐based neural probes incorporating micro‐LEDs on silicon substrates have demonstrated efficacy in vivo for simultaneous stimulation and recording,^[^ ^18^, ^19^, ^20^ ^]^ their mechanical mismatch with the soft, viscoelastic nature of brain tissue often results in glial scarring, micromotion‐induced damage,^[^ ^21^, ^22^, ^23^, ^24^ ^]^ and eventual degradation in signal quality.^[^ ^25^ ^]^ Moreover, micro‐LEDs are prone to localized heat accumulation during high‐frequency use, further compromising biocompatibility and posing a risk of thermal injury to surrounding tissue.^[^ ^26^, ^27^, ^28^ ^]^ In response, recent advances have explored polymer‐based flexible substrates for integrating micro‐LEDs,^[^ ^27^ ^]^ aiming to reduce mechanical mismatch and improve tissue conformability.^[^ ^3^ ^]^ However, these soft platforms still face critical long‐term reliability issues: common materials such as polyimide, PDMS, or parylene are susceptible to water uptake and hydrolytic degradation under physiological conditions.^[^ ^29^, ^30^, ^31^, ^32^, ^33^ ^]^ Over time, these processes may cause microcrack formation, delamination, or dielectric breakdown in encapsulation layers,^[^ ^34^ ^]^ especially around embedded inorganic elements like micro‐LEDs. These failure modes can result in leakage currents, device delamination, and localized electrochemical injury to adjacent neural circuits,^[^ ^33^, ^35^, ^36^ ^]^ compromising both device function and safety by potentially stimulating off‐target regions or inducing neurotoxicity.^[^ ^37^ ^]^ These material and electrical stability challenges represent major obstacles to deploying soft optoelectronic implants for chronic, closed‐loop neuromodulation, where long‐term reliability, safety, and high‐fidelity performance are essential. Compounding these limitations, gene delivery remains a critical bottleneck. Although adeno‐associated viruses (AAVs) dominate current optogenetic protocols due to their high transduction efficiency,^[^ ^38^ ^]^ they pose serious risks including immunogenicity, off‐target expression, and biosafety issues related to long‐term gene integration,^[^ ^39^ ^]^ and further constrained by stringent regulatory barriers that limit scalability and individualization. Additionally, conventional systems often isolate gene delivery, optical stimulation, and electrophysiological recording into separate hardware modules, an architectural fragmentation that increases surgical complexity, hinders precise spatial co‐localization, and reduces the overall efficacy and integration of the neuromodulation interface.

To overcome the limitations of current optogenetic systems, there is an urgent need for a unified, miniaturized, and chronically stable neural interface that integrates non‐viral gene delivery, NIR‐mediated fiberless intracranial optogenetic stimulation, and real‐time electrophysiological sensing into a single implantable platform. Such systems must avoid invasive light guides, minimize immune and thermal damage, and maintain long‐term functionality for closed‐loop, cell‐type‐specific neuromodulation. Remote optogenetic control using upconversion nanoparticles (UCNPs) offers a promising solution by converting near‐infrared (NIR) light to visible blue light via anti‐Stokes shift,^[^ ^40^, ^41^, ^42^, ^43^ ^]^ enabling deep brain stimulation without optical fibers.^[^ ^44^, ^45^ ^]^ Encapsulation in biocompatible hydrogels like GelMA improves stability and tissue integration,^[^ ^46^, ^47^ ^]^ while coupling UCNPs with plasmonic gold inverse opal (AuIO) substrates enhances upconversion efficiency through localized surface plasmon resonance (LSPR),^[^ ^48^ ^]^ allowing lower remote NIR intensities and reducing thermal risk.^[^ ^48^, ^49^, ^50^ ^]^ In parallel, non‐viral gene delivery systems have emerged as safer and more controllable alternatives to viral vectors.^[^ ^51^, ^52^, ^53^ ^]^ Among them, polyethylenimine (PEI)‐based nanocarriers exhibit high nucleic acid condensation efficiency,^[^ ^54^ ^]^ tunable release kinetics, and low immunogenicity.^[^ ^54^ ^]^ By coupling PEI with targeting ligands such as neurotensin (NT)^[^ ^55^, ^56^ ^]^ and using electroporation‐assisted membrane permeabilization, these carriers can achieve highly localized and transient gene expression,^[^ ^57^, ^58^, ^59^ ^]^ suitable for closed‐loop applications.^[^ ^60^, ^61^ ^]^ However, current devices typically segregate gene delivery, optical stimulation, and electrophysiological readout into separate modules, increasing surgical complexity and limiting spatial precision.

In this study, we present a multifunctional, biocompatible neural interface for minimally invasive, spatiotemporally precise, closed‐loop neuromodulation. Built on a 3D AuIO microelectrode, our platform integrates non‐viral delivery of AAV‐derived plasmid, fiberless NIR‐driven intracranial optogenetic stimulation, and electrophysiological recording. The porous AuIO structure serves as both a high‐capacity gene reservoir loaded with NT‐conjugated PEI–ChR2 plasmid (AAV backbone) nanocomplexes, and a light amplifier integrated with UCNPs‐encapsulated GelMA. A single implantation enables localized electroporation‐based opsin delivery and remote stimulation using collimated NIR light, eliminating the need for fiber optics. Enhanced electrochemical properties and LSPR‐coupled UCNP upconversion allow efficient blue light emission under low‐power NIR. In vivo validation in the mouse hippocampal dentate gyrus (DG) confirms effective gene transfection, NIR‐driven neuronal activation, and high‐fidelity multi‐unit recording. Collectively, this work establishes a versatile, minimally invasive neural interface that overcomes the limitations of viral vectors and optical fibers, offering a promising solution for next‐generation neuromodulation technologies with closed‐loop capability.

## 2 Results and Discussion

**Scheme 1:**
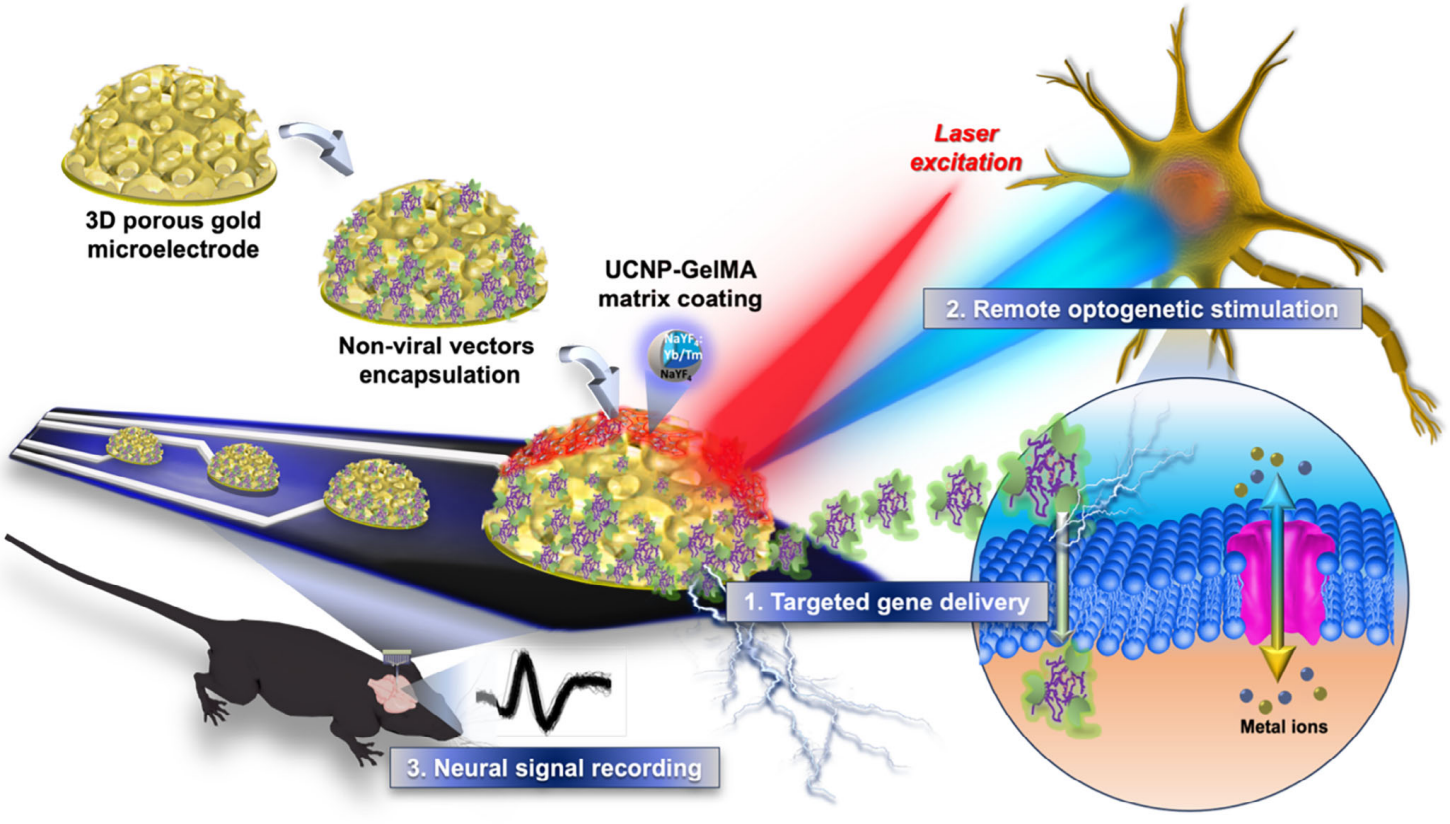
Overview of a multifunctional 3D AuIO electrode array with closed‐loop neuromodulation capability in vivo. The system integrates three core functionalities: (1) targeted gene delivery, (2) optogenetic stimulation, and (3) neural signal recording, all within a unified platform. (1) The 3D AuIO structures are fabricated via templated electrodeposition and offer a high surface area for the immobilization of NT‐PEI complexes carrying the ChR2 plasmid based on an AAV backbone. (2) UCNPs are embedded in a GelMA hydrogel matrix and inkjet‐printed onto the electrode surface, enabling remote activation of ChR2 by converting near‐infrared excitation into visible light. (3) Neural signals are recorded in real time through the same electrode array, supporting the implementation of a closed‐loop interface. The AuIO nanostructure further enhances the optical output of UCNPs through localized surface plasmon resonance, thereby improving the efficiency of optogenetic stimulation. Together, these elements provide the essential functionality for future closed‐loop neuromodulation systems.

**Scheme** **1** presents the integrated design of a multifunctional 3D AuIO spatiotemporal electrode array enabling closed‐loop neuromodulation. The platform is constructed via a sequential fabrication process beginning with the electrophoretic self‐assembly of polystyrene (PS) microspheres on microelectrode surfaces, forming hemispherical templates. Gold (Au) is backfilled via electrodeposition, and removal of the PS template yields porous 3D AuIO nanostructures. These microporous electrodes provide a high surface area for efficient loading of NT‐PEI‐based, non‐viral vectors carrying AAV‐derived and channelrhodopsin‐2 (ChR2) plasmids, which are delivered intracellularly via electroporation.

To enable wireless optogenetic stimulation, UCNPs embedded in GelMA hydrogel are precisely patterned onto the AuIO microelectrodes using in‐jet printing. Upon 980 nm NIR excitation, UCNPs convert the light to visible wavelengths, activating the membrane‐bound ChR2 opsins and eliciting neuronal responses.

The integrated platform supports closed‐loop modulation by combining three core functionalities: 1) targeted gene delivery, 2) remote optogenetic stimulation, and 3) real‐time neural signal recording. The recorded electrophysiological signals can be processed and analyzed in real‐time to dynamically adjust stimulation parameters via an external current stimulator, forming a feedback‐controlled neuromodulatory system. Additionally, the 3D AuIO architecture not only maximizes gene vector loading but also enhances the upconversion efficiency of UCNPs through LSPR, further improving light‐driven activation precision and efficacy.

### 2.1 Development of Biocompatible Non‐Viral Gene Vector

**Figure 1:**
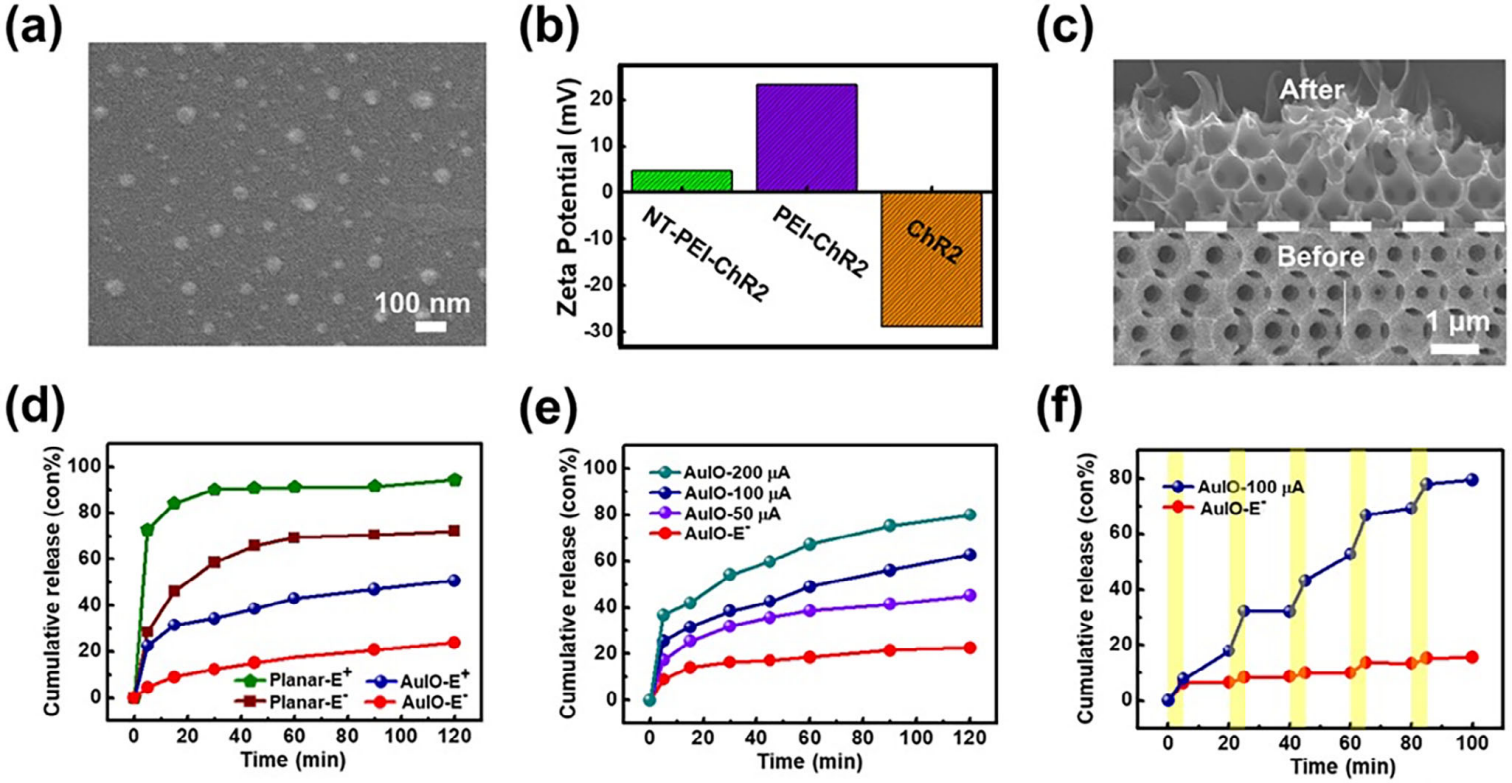
(a) Representative SEM images showing the spherical morphology and uniform dispersion of the NT‐PEI‐ChR2 nanocomplex. The complex was formed by self‐assembly of NT‐PEI with ChR2 plasmid DNA, demonstrating nanoscale dimensions suitable for cellular uptake. (b) Zeta potential of NT‐PEI‐ChR2, PEI‐ChR2, and ChR2. (c) Cross‐sectional SEM image of the electrode with 3D AuIO structure on ITO substrate before and after loading with the NT‐PEI‐ChR2 nanocomplex. The porous framework (bottom, “Before”) provides interconnected opal voids (≈800 nm) for accommodating the gene delivery complex. After loading (top, “After”), the pore walls are coated with a polymer‐rich layer exhibiting a tentacle‐like morphology, indicating successful infiltration and attachment of the NT‐PEI‐ChR2 within the 3D scaffold. (d) Cumulative release profiles of NT‐PEI‐ChR2 genes from planar Au and 3D AuIO structures with or without 100 µA continuous electrical stimulation (1 Hz, 250 ms pulse width). “Planar‐E^+^” and “Planar‐E^−^” indicate planar Au with and without stimulation; “AuIO‐E^+^” and “AuIO‐E^−^” indicate 3D AuIO with and without stimulation. (e) Cumulative NT‐PEI‐ChR2 release profiles of genes loaded in 3D AuIO structure apply with continuous electrical stimulation at 1 Hz, 250 ms duration with different current intensities: 0, 50, 100, and 200 µA, respectively. (f) Cumulative stepwise release profiles of NT‐PEI‐ChR2 loaded in the 3D AuIO structure under cyclic electrical stimulation. A biphasic current of 0 and 100 µA was alternately applied for 5 min, followed by a 15 min rest period, with a frequency of 1 Hz and a pulse width of 250 ms. The electrical stimulation periods are highlighted by yellow bands. The AuIO‐E^−^ group represents the control without electrical stimulation.

Non‐toxicity and high transfection efficiency are two critical requirements for an effective gene delivery system. NT, a neuropeptide known for its neuro‐targeting and biocompatibility properties, was chemically conjugated to PEI to enhance cellular uptake and transfection efficiency, as supported by our previous report.^[^ ^62^ ^]^ The resulting NT‐PEI served as a non‐viral vector to deliver Channelrhodopsin‐2 plasmid DNA (ChR2) effectively while maintaining low cytotoxicity. The resultant nanocomplex, called NT‐PEI‐ChR2, exhibits a uniformly dispersed, spherical nanostructure with an average size in the nanometer range, indicating successful self‐assembly of the NT‐PEI with ChR2 plasmid DNA. (**Figure** **1**a; Figure S1, Supporting Information). The agarose gel retardation assay demonstrated an *N/P* ratio of 0.05k, defined as the molar ratio of the amine groups in cationic NT‐PEI to the phosphate groups in ChR2 plasmid DNA, was sufficient to fully bind and immobilize ChR2, confirming complete complexation and formation of a stable spherical nanostructure (Figure S2, Supporting Information). The biocompatibility of NT‐PEI‐ChR2 was further validated using a CCK‐8 assay in PC‐12 cells (Figure S3, Supporting Information). The cell viability results confirmed that the NT‐PEI‐ChR2 complex exhibited minimal cytotoxicity, supporting its suitability for safe gene delivery applications. Meanwhile, the NT‐PEI‐ChR2 retained a positive zeta potential (Figure 1b), which facilitates electrostatic attraction to negatively charged cell membranes to enhance cellular uptake and transfection efficiency.

### 2.2 Electrically Controlled Gene Delivery via 3D AuIO Architecture

In this study, an AuIO architecture was developed in the microelectrodes to effectively capture and protect NT‐PEI‐ChR2. The NT‐PEI‐ChR2 complex was loaded into the interconnected nanoscale cavities (≈700 nm) of the 3D AuIO at a surface concentration of 120 ng/cm^2^. Owing to its high theoretical porosity (≈74%), the AuIO scaffold offered substantial internal volume to encapsulate and physically restrict the loaded gene vectors. Cross‐sectional SEM imaging (Figure 1c) confirmed a distinct morphological transition after gene loading: a polymer‐rich, tentacle‐like coating was observed along the pore walls, suggesting effective filling of the nanostructured scaffold. This was further validated by EDX elemental mapping (Figure S4, Supporting Information), showing uniform distribution of carbon (*C*), nitrogen (*N*), and phosphorus (*P*) within the void network, corresponding to the NT‐PEI and ChR2 components.

Subsequently, we designed a comparative study using two electrode architectures: i) a planar Au nanofilm structure deposited on ITO substrates, and ii) the engineered 3D AuIO porous architecture. This design with identical surface material chemical properties and electrochemical compatibility, ensuring that any observed differences in gene retention and release behavior could be attributed to differences in surface topography and structural porosity, rather than variations in surface tension or material chemistry. To exclude the possibility that observed release kinetics were driven by intrinsic material properties, we compared gene retention performance against the planar Au surface under identical loading conditions (Figure S5, Supporting Information). When applied to the planar structure, the gene complexes exhibited rapid dissociation, particularly under electrical stimulation (Planar‐E^+^). In contrast, the 3D AuIO structure significantly restrained passive leakage (AuIO‐E^−^), indicating an enhanced physical entrapment effect due to the porous geometry.

We then investigated the electrically triggered release behavior. As shown in Figure 1d, the AuIO‐E^+^ group enabled sustained release over 3 h under continuous electrical pulsing, demonstrating the system's suitability for controlled and prolonged gene delivery. To further assess the system's programmability, we evaluated the release profiles under varying stimulation intensities (50, 100, and 200 µA; Figure 1e). The cumulative release exhibited a clear dependence on the applied current, with the 200 µA group achieving a release concentration of 192 ng/mL, approximately fourfold higher than the passive diffusion group (AuIO‐E^−^). This demonstrates that both the duration and amplitude of electrical input can be used to modulate gene release dosage, providing a powerful temporal control mechanism. Collectively, these findings support the conclusion that the 3D AuIO structure not only enhances loading capacity and protection of gene complexes but also enables electrically tunable release,^[^ ^63^, ^64^ ^]^ offering significant advantages for on‐demand gene delivery in neuromodulation of our lab‐designed MEA chip^[^ ^65^ ^]^ and neural electrode array.^[^ ^66^ ^]^

To further investigate the release kinetics and underlying mechanisms, we analyzed the release profiles using the power law model^[^ ^67^ ^]^ (Tables S1 and Table S2, Supporting Information). This analysis revealed distinct gene release behaviors among the different groups. The Planar‐E^−^group demonstrated *super case II transport* (N = 1.10, Table S1, Supporting Information), indicating that polymer relaxation dominated the rapid release process. In contrast, the AuIO‐E^−^ group exhibited anomalous transport (N = 0.51, Table S1, Supporting Information), suggesting a combined influence of diffusion and polymer relaxation, constrained by the physical hindrance within the 3D AuIO matrix. For the AuIO‐E^+^ group, the release exponent (N < 0.5, Table S2, Supporting Information) from fitting the power law equation confirmed Fickian diffusion, and a further decrease in release exponent (N) with increasing electrical intensity indicated that higher current levels enhanced polymer chain relaxation dynamics, thereby accelerating gene release.^[^ ^68^ ^]^

To assess the system's capability for temporal and programmable control, a stepwise release experiment was performed by applying a 5 min actuation of 100 µA current, followed by a 15 min resting phase (Figure 1f). This specific current intensity was chosen because it aligns with the typical stimulation amplitude used in safe and effective neuromodulation,^[^ ^66^ ^]^ ensuring compatibility with neural interface applications while providing sufficient energy to modulate polymer relaxation and release rates in a controlled manner. The results demonstrated that gene release could be precisely toggled on and off with the applied current, enabling electrically controlled, on‐demand NT‐PEI‐ChR2 gene delivery appropriate for neuromodulation contexts. This highlights the promise of this system for future bioelectronic therapies requiring synchronized gene release in response to neural stimulation parameters.

### 2.3 Localized Electroporation and Enhanced Electrochemical Interface via 3D AuIO Microarchitecture

In addition to releasing the gene, electrical stimulation pulses of our gene delivery system facilitated electroporation, which enhanced gene delivery by creating temporary pores on the cell membrane, leading to improve the transfection efficiency with less cell damage.^[^ ^69^ ^]^ Our unique microporous architecture of the 3D AuIO modification provides an expanded electroactive surface area, which substantially increases the non‐faradaic double‐layer capacitance by approximately 7.9‐fold and reduces the impedance by about 117.5‐fold compared to the conventional planar electrode configuration (Figure S6a,b, Supporting Information). Such pronounced improvements in interfacial capacitance and impedance characteristics are critical for enhancing the signal‐to‐noise ratio and fidelity of neural signal acquisition, as previously emphasized in neural interface studies.^[^ ^70^, ^71^, ^72^ ^]^ Moreover, the facilitated ion transport and rapid charge transfer kinetics inherent to the porous structure enable more efficient and localized energy delivery, which is particularly beneficial for optogenetic applications requiring instantaneous energy release, such as electroporation‐assisted neural modulation.^[^ ^73^, ^74^, ^75^ ^]^ Overall, these findings demonstrate that micro/nano‐structured 3D porous Au architecture is a highly effective strategy to optimize our neural implant device for both recording and stimulation functionalities.

**Figure 2:**
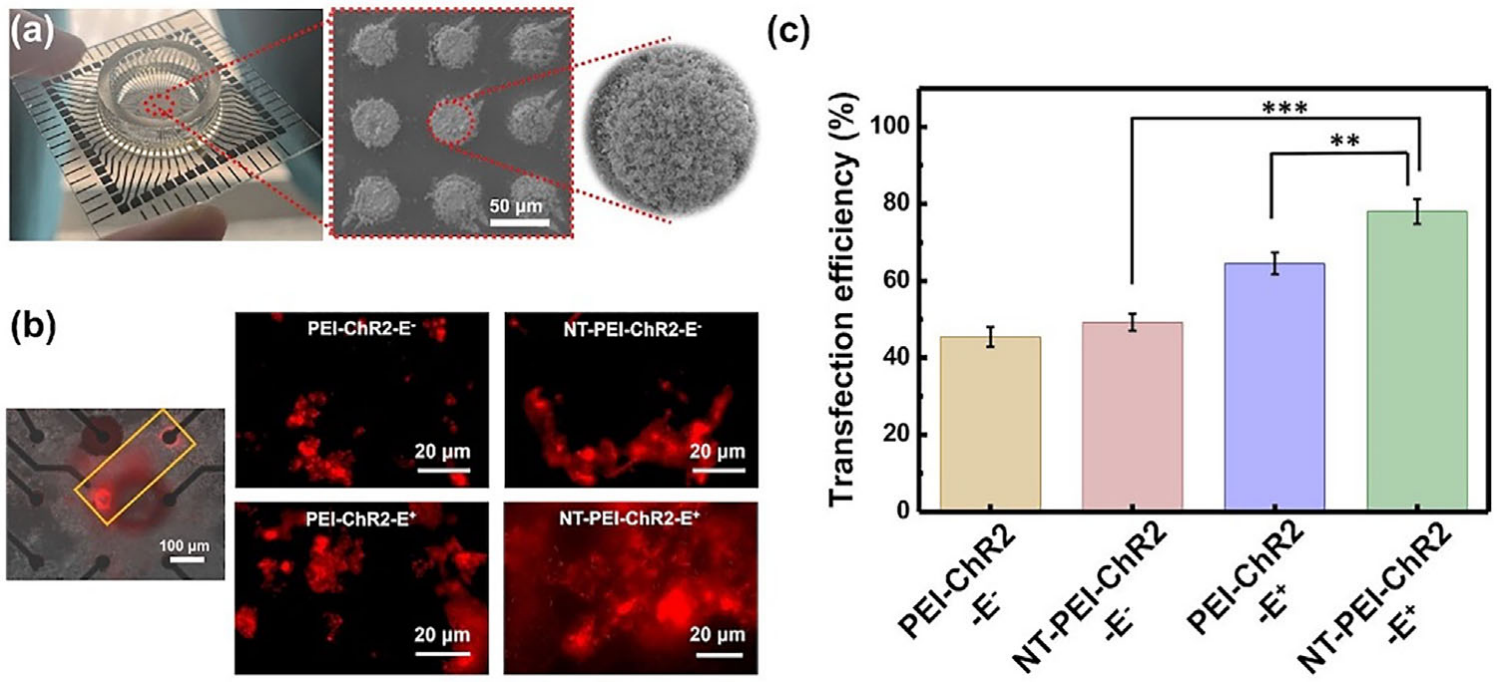
Localized electroporation‐enabled gene delivery using 3D AuIO‐modified MEA. (a) Photograph and SEM of the MEA chip showing uniform integration of 3D AuIO nanostructures on each microelectrode. (b) Left: Fluorescence microscopy confirming site‐specific mCherry expression between paired microelectrodes in the orange block. Right: Representative images of neuronal cells at 48 h post‐transfection under four different conditions: PEI‐ChR2‐E^−^, NT‐PEI‐ChR2‐E^−^, PEI‐ChR2‐E^+^, and NT‐PEI‐ChR2‐E^+^. (c) Quantification of transfection efficiency by flow cytometry. NT‐modified complexes and electrical stimulation synergistically enhance localized gene delivery, achieving highest efficiency in the NT‐PEI‐ChR2‐E^+^ group (*n* = 3, mean ± SD).

**Figure 3:**
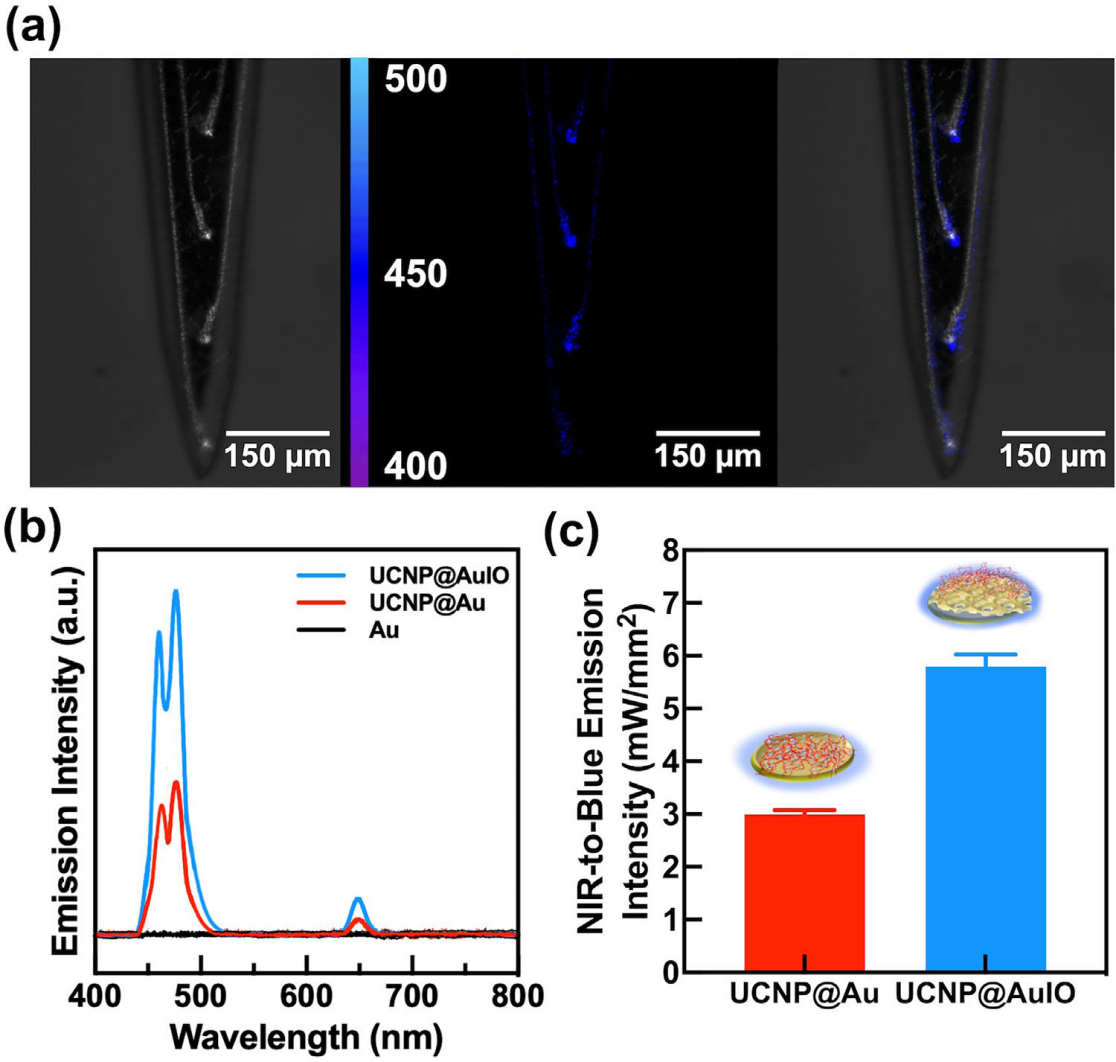
Upconversion blue emission behavior of GelMA‐based UCNPs printed on 3D AuIO‐modified microelectrodes under NIR excitation. (a) Optical and fluorescence composite images of the neural electrode array at different NIR illumination conditions. Left: Bright‐field image of the array with GelMA‐based UCNPs printed on 3D AuIO microelectrodes via aerosol jet printing under NIR‐off condition, showing no observable upconversion emission. Middle: Under 50 mW 980 nm NIR illumination, distinct localized blue upconversion emission (400–500 nm) emerges from the UCNPs printed on the 3D AuIO microelectrodes. Right: Alignment of the fluorescence emission map with the underlying microelectrode architecture confirms that the blue light emission originates specifically from the UCNPs on the microelectrode sites, maintaining strong spatial confinement. (b) Emission spectra of different microelectrode configurations under 50‐mW 980 nm NIR excitation, measured using a micro‐Raman system coupled with a CCD‐based spectrometer. The UCNP@3D AuIO microelectrodes (blue line) exhibit substantially enhanced blue light emission (450–500 nm) compared to UCNP@Au microelectrodes (red line), while bare Au microelectrodes (black line) show negligible emission in the same spectral range. (c) Quantitative analysis of NIR‐to‐blue emission intensity. The integrated blue emission power density (450–500 nm) from UCNP@3D AuIO microelectrodes reaches 5.81 mW/mm^2^, which is significantly higher than that from UCNP@Au microelectrodes (2.93 mW/mm^2^). The enhancement is attributed to the LSPR effect provided by the 3D nanostructured AuIO architecture. Data are presented as mean ± SD. *Note: Bare Au microelectrodes are excluded from the bar graph due to their negligible blue emission under the measurement conditions*.

Achieving precise spatiotemporal control in complex brain regions remains a major challenge for optogenetic gene delivery. To address this, we developed a MEA^[^ ^65^ ^]^ platform modified with 3D AuIO nanostructures to enable localized electroporation‐mediated gene transfer. The nanostructured AuIO architecture not only enhances the local electric field at each microelectrode but also provides high surface area for efficient gene complex loading. As shown in **Figure** **2**a and 3D AuIO structures were successfully fabricated onto the MEA chip, and localized electroporation was applied to achieve site‐specific delivery. Fluorescence microscopy revealed confined expression of mCherry‐labeled genes within targeted microelectrode regions (Figure 2b, left), validating the spatial precision of this approach. This precise localization is attributed to the microscale electric fields focused by the AuIO‐enhanced electrodes, enabling high‐resolution delivery control.

To investigate the synergistic effects of NT‐mediated targeting and electroporation, four experimental groups were tested using PEI‐ChR2 or NT‐PEI‐ChR2 plasmid complexes with or without electrical stimulation. The images of red fluorescence mCherry‐labeled cells of each group at 48 h post‐transfection are shown in Figure 2b (right panel). These images were consistent with the flow cytometry results (Figure 2c and Figure S7, Supporting Information), showing transfection efficiency was 45%, 49%, 64% and 78% in PEI‐ChR2‐E^−^, NT‐PEI‐ChR2‐E^−^, PEI‐ChR2‐E^+^, and NT‐PEI‐ChR2‐E^+^ groups, respectively. These results demonstrated the higher transfection efficiency of NT‐PEI‐ChR2 compared to PEI‐ChR2 because of the NT‐enhancement of neuron‐targeting effect. Moreover, groups which subjected to electrical stimulation (E^+^) exhibited increased transfection efficiency, confirming that the electroporation by electrical stimulation significantly enhances cell uptake. It is worth noting that the NT‐PEI‐ChR2‐E^+^ group achieved an impressive value of transfection efficiency, attributed to electrostatic attraction and the targeting ability, which increased the concentration of the non‐viral vector near the negatively charged cell surface, thus improving transfection efficiency in specific regions.

### 2.4 Plasmon‐Enhanced Upconversion and Hydrogel Stabilization for Remotely Optogenetic Modulation

To overcome the inherent limitations of blue light (475 nm) in deep brain stimulation, particularly its poor tissue penetration and scattering, UCNPs were employed as a wireless optogenetic light transducer, capable of converting 980‐nm NIR light into visible emission to activate ChR2 opsins.^[^ ^76^, ^77^, ^78^ ^]^ To ensure mechanical stability and adhesion of the UCNP coating during and after implantation, two coating strategies were compared: a water‐based UCNP solution and a UV‐crosslinked GelMA‐based UCNP hydrogel coating, both applied on ITO‐based AuIO‐modified microelectrodes. As shown in Figure S8a and Video S1 (Supporting Information), the water‐based UCNP coating was easily displaced during simulated penetration into an agarose‐based brain phantom. In contrast, the GelMA‐based UCNP coating retained its structural integrity and remained firmly attached to the AuIO surface following insertion. This observation demonstrates that the GelMA matrix significantly improves the mechanical stability of the UCNP layer, preventing delamination and ensuring the coating remains functional under brain‐implantation conditions. The successful implementation of this coating strategy is schematically illustrated in Figure S8b (Supporting Information), highlighting the coaxial UV‐crosslinking process used to immobilize the UCNP‐hydrogel on the electrode.

To quantitatively assess the plasmonic enhancement effect introduced by the 3D AuIO nanostructure, we first compared the upconversion luminescence (UCL) characteristics between UCNP@AuIO and UCNP@planar Au electrodes. GelMA‐based UCNP hydrogel coatings were uniformly deposited and UV crosslinked on both electrode types on the ITO substrate. As shown in Figure S9a (Supporting Information), the UCNP@AuIO group exhibited a significantly enhanced blue emission peak at ≈475 nm, with a peak intensity of ≈450 a.u. and an integrated spectral area of ≈8500 a.u. in the 465–485 nm range. In contrast, the UCNP@planar Au electrode yielded only ≈230 a.u. peak intensity and ≈4200 a.u. integrated area, confirming an approximately 2‐fold enhancement in blue UCL, attributed to LSPR effects from the curved, porous 3D AuIO structure.^[^ ^79^, ^80^, ^81^ ^]^

To assess functional relevance for optogenetics, we conducted output power measurements under increasing 980 nm NIR excitation (Figure S9b, Supporting Information). At 50 mW NIR input power, the UCNP@AuIO electrode on the ITO structure produced a blue emission power density of ≈5.86 mW/mm^2^. In contrast, UCNP@planar Au electrodes generated a ≈2.47 mW/mm^2^. Notably, both values surpass the commonly accepted threshold for ChR2 activation (≈1–5 mW/mm^2^),^[^ ^82^, ^83^, ^84^ ^]^ indicating that even at relatively low NIR power levels with reducing localized heating effects,^[^ ^85^, ^86^ ^]^ UCNP@AuIO can safely and effectively elicit neural activation. The dual benefit of LSPR‐enhanced light conversion and GelMA‐induced mechanical stabilization makes this configuration suitable for wireless in vivo stimulation.

### 2.5 Advanced UCNP Printing and Optical Validation on 3D AuIO Neural Electrode Arrays

To enable spatially confined and optogenetically effective light emission, we adopted aerosol jet printing to precisely deposit GelMA‐based UCNP inks onto individual sites of the 3D AuIO‐modified neural electrode array. This approach offered micrometer‐scale precision, enabling the fabrication of uniform coatings on electrodes with ≈18 µm diameters without spreading to adjacent regions. As shown in Figure S10a (Supporting Information), GelMA‐based UCNP inks demonstrated superior patterning fidelity and coating integrity compared to conventional water‐based dispersions, which formed irregular, discontinuous trails due to poor substrate adhesion and instability under printing shear.

The compatibility of this approach with 3D microarchitectures is visualized in Figure S10b (Supporting Information), which depicts three sequential states of the electrode: a bare planar electrode, a 3D AuIO‐coated electrode, and finally a UCNP‐GelMA composite integrated on the 3D AuIO scaffold. The conformality and intimate coverage of UCNPs across the porous AuIO surface offer mechanical robustness while preserving nanoscale topography essential for light field enhancement.

To verify that the NIR‐converted blue light on these printed electrode array electrodes is sufficient for optogenetic stimulation, a customized optical measurement system was developed (Figure S11, Supporting Information). This system incorporates a Y‐type bifurcated optical fiber that interfaces a 50× objective lens with two components: a 980‐nm NIR laser diode and a CCD‐coupled spectrometer. This dual‐function setup enables simultaneous NIR excitation and emission light detection at the microscale. The NIR output is regulated by a digital laser driver, and a calibrated photodiode sensor connected to a power meter quantifies the absolute emission power across 400–1100 nm.

Functionally, this architecture facilitated localized upconversion upon NIR exposure. Under pulsed 980 nm laser stimulation (1 Hz), UCNP@AuIO‐modified microelectrodes produced site‐specific blue emission, as shown in **Figure** **3**a and dynamic emission in Video S2 (Supporting Information). Spectral quantification (Figure 3b) confirmed a marked increase in emission at ≈475 nm, correlating with optogenetically relevant wavelengths for ChR2 activation.

Quantitative analysis (Figure 3c) further demonstrated that under 50 mW NIR illumination, the same condition validated in Figure S9b (Supporting Information), UCNP@AuIO microelectrodes generated an estimated blue‐light power density of ≈5.81 mW/mm^2^, while the UCNP@Au electrodes achieved ≈2.93 mW/mm^2^. These values both exceed the ≈1 mW/mm^2^ activation threshold for ChR2 opsins, affirming the suitability of our wireless stimulation approach.^[^ ^82^ ^]^ Notably, this ≈2× enhancement observed at the microscale (Figure 3c) closely parallels the trend seen at the millimeter‐scale electrodes (Figure S9b, Supporting Information), where UCNP@AuIO also demonstrated approximately doubled the blue emission output compared to UCNP@planar Au under 50 mW NIR excitation. This consistent performance across different scales, from millimeter‐scale photodetector substrates to microscale neural electrodes, demonstrates the scalable efficiency and robustness of the 3D AuIO‐enhanced architecture. The reproducibility of this enhancement confirms the central role of the 3D AuIO structure in facilitating strong localized plasmonic coupling. Whether implemented on ITO‐glass electrodes or confined within 15 µm microelectrodes of a neural electrode array, the AuIO scaffold consistently enhances light conversion efficiency, enabling robust optogenetic modulation under low‐intensity NIR stimulation both ex vivo and in vivo.

### 2.6 Ex vivo Optogenetic Activation and Recording Using UCNP@3D AuIO‐Modified MEAs

**Figure 4:**
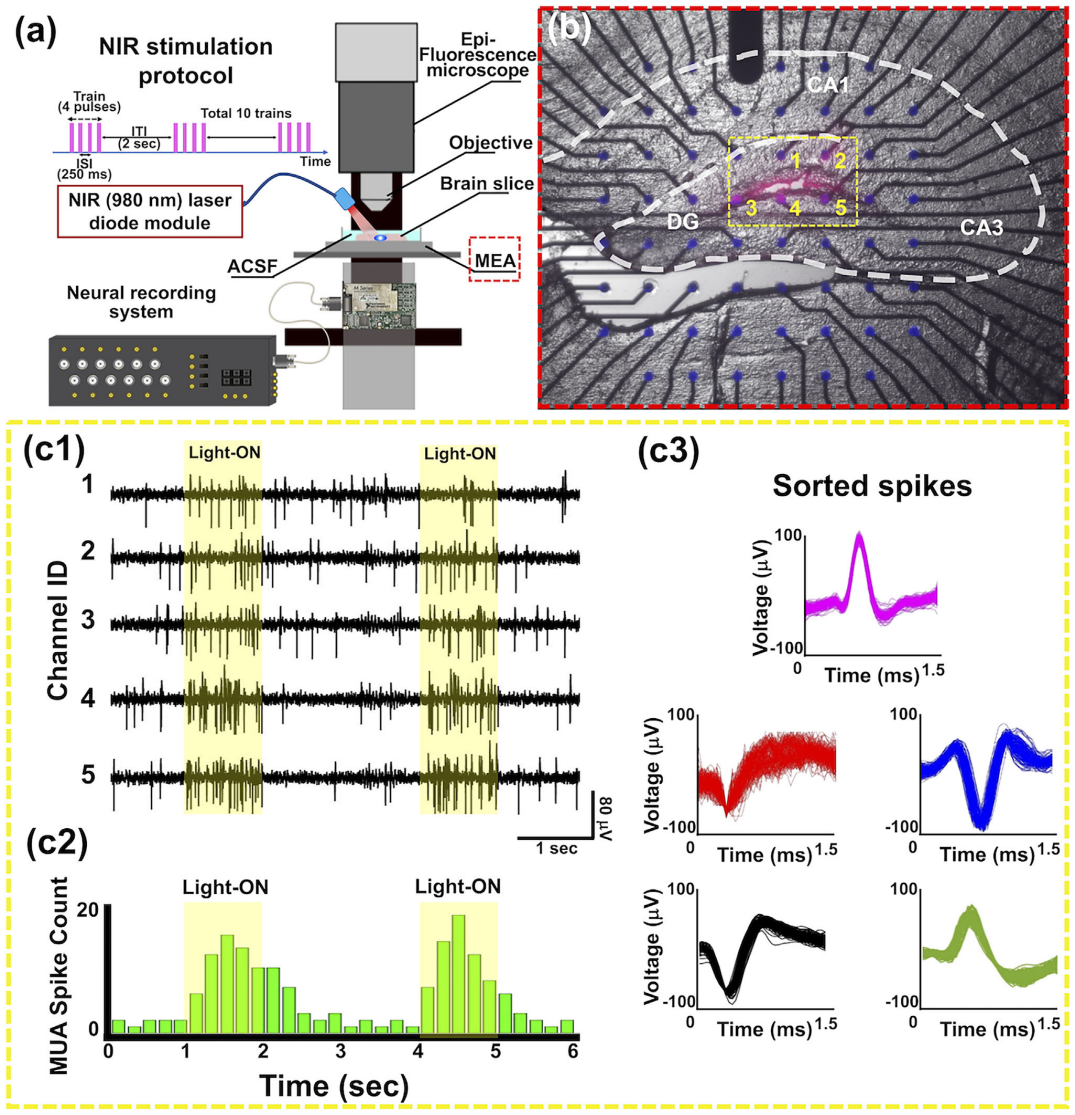
NIR light stimulation of acute hippocampal slices on MEAs excited neural activation ex vivo. (a) Schematic of the experimental setup illustrating remote optical stimulation of ChR2‐mCherry‐expressing region in hippocampal DG using UCNP@3D AuIO modified MEA platform. (b) Fluorescence microscopy combined with DIC imaging was used to visualize the acute cortico‐hippocampal slice placed on the MEA device. The red dashed box highlights the MEA device corresponding to the area marked in panel (c). The ChR2‐mCherry‐expressing tissue region was identified and marked with a yellow dotted box. This region overlies five UCNP@3D AuIO modified microelectrodes, which were used for remote optogenetic stimulation by converting NIR light to blue light via UCNPs. c1–c3) Neural activation was recorded through the five modified microelectrodes positioned beneath the ChR2‐mCherry‐expressing tissue. Upon NIR irradiation, the UCNPs emitted blue light that stimulated the ChR2‐expressing hippocampal neurons, resulting in evoked MUA. The yellow dotted boxes in these panels correspond to the same tissue region indicated in panel (b). The panels present spike train recordings and quantitative analyses, confirming that UCNPs integrated with 3D AuIO on the MEA can achieve effective light‐induced neural stimulation ex vivo, supporting their potential for future in vivo optogenetic applications.

In order to achieve proof of concept, we conducted an ex vivo optogenetic stimulation and electrophysiological validation using UCNP@3D AuIO‐modified MEA.^[^ ^17^ ^]^ The UCNP@3D AuIO‐modified MEA was engineered to enable localized transduction of NIR light into visible blue emission, thereby allowing wireless and spatially precise activation of opsin‐expressing neurons. To implement this functionality, a 980 nm pulsed‐NIR laser was positioned 30 mm above the MEA surface to deliver broad, unfocused illumination across the tissue–MEA interface (**Figure** **4**a). A burst‐mode photostimulation protocol was employed to emulate physiologically relevant neural activation patterns.

Acute ChR2‐mCherry expression cortico‐hippocampal brain slices (≈500 µm thick) was precisely positioned atop the UCNP@3D AuIO‐modified MEA (Figure 4b). Identification of the ChR2‐expressing neuronal tissue within the DG subregion of the hippocampus was accomplished through combined infrared differential interference contrast (IR‐DIC) and fluorescence imaging for mCherry detection. This dual‐modality imaging ensured accurate spatial correlation between the targeted opsin‐positive region and the underlying UCNP‐modified microelectrodes.

Upon NIR irradiation, the embedded UCNPs efficiently converted low‐energy photons into localized blue luminescence, which was sufficient to activate the ChR2 expressed in hippocampal pyramidal neurons. Electrophysiological recordings obtained from the five designated UCNP‐modified microelectrodes revealed clear MUA, temporally locked to the NIR photostimulation intervals (Figure 4c). Spike sorting analyses further confirmed the presence of optically evoked responses, thereby validating the functional transduction capacity of the UCNP@3D AuIO interface.

### 2.7 In Vivo Wireless Optogenetic Control Using UCNP@3D AuIO‐Modified Neural Electrode Array

**Figure 5:**
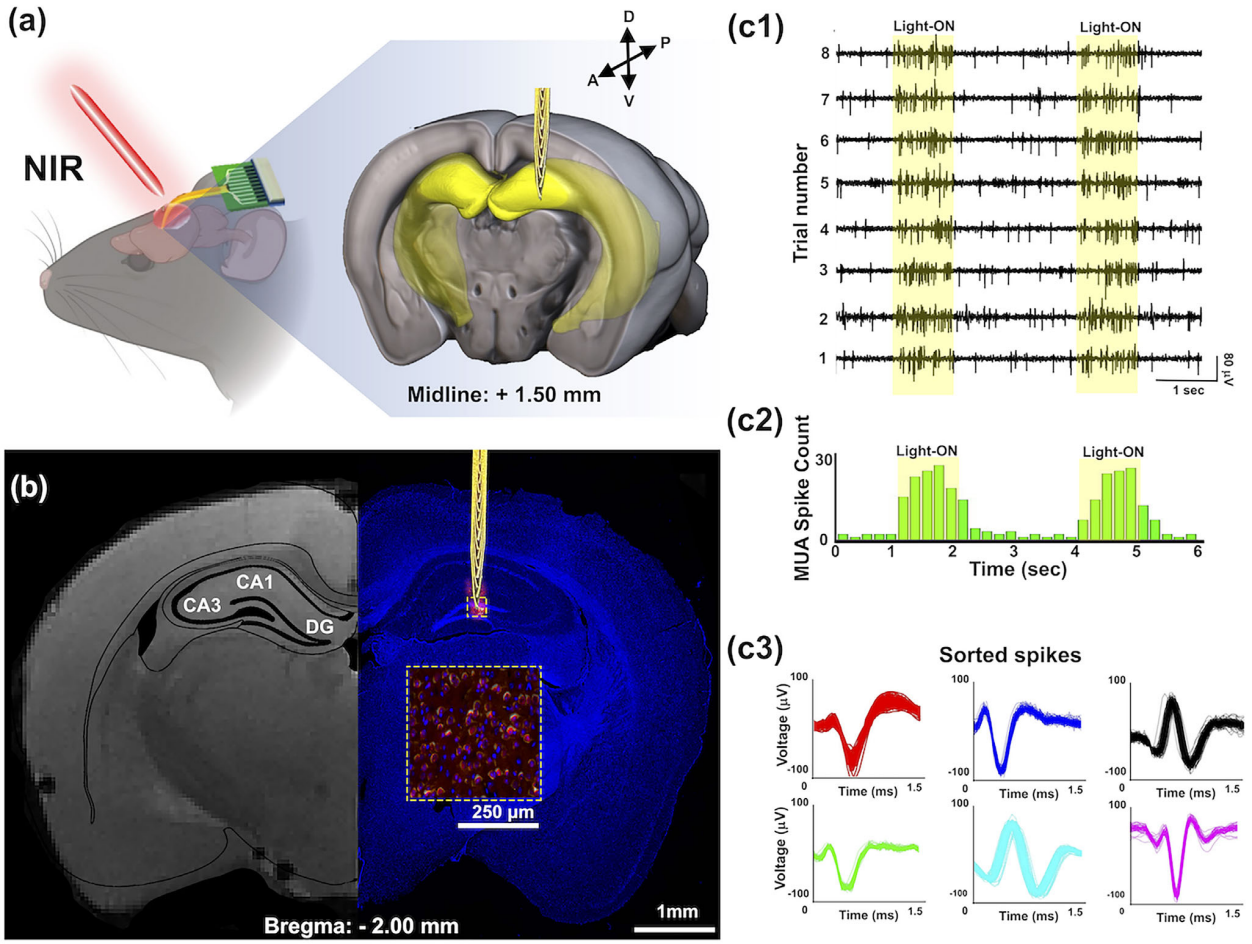
In vivo remote NIR light stimulation elicited neural activity in the hippocampal DG region. (a) Schematic illustration of the configuration of the neural electrode array and corresponding surgical placement for targeted opsin gene delivery and subsequent NIR‐responsive photostimulation in the DG of mice. The 16‐channel neural electrode array was sequentially functionalized using aerosol jet printing: GelMA‐encapsulated UCNPs were deposited on Ch #2 and Ch #3 for optical transduction, followed by localized coating of the PEI−NT−ChR2‐mCherry nanocomplex onto Ch #1 and Ch #4 for electroporation‐mediated gene transfection. This configuration enabled one‐step surgical implantation to achieve spatially confined gene delivery and fiberless intracranial optogenetic activation using 980 nm NIR excitation. (b) Confocal fluorescence imaging demonstrates localized ChR2–mCherry expression within the hippocampal DG seven days after implantation and electroporation, confirmed by immunohistochemistry. DAPI (blue) labels cell nuclei, while mCherry (red) indicates successful opsin expression. Expression is concentrated near the probe tract at channels Ch#1 and Ch#4, with partial signal observed in the CA1 region, reflecting spatially confined gene delivery consistent with the electric field distribution and the orientation of neural electrode array. A photomicrograph of a coronal brain section at AP −2 mm relative to bregma confirms the in situ placement of the implanted neural electrode array. Magnified fluorescence images (yellow dotted box) highlight strong ChR2–mCherry expression specifically within the DG, while wide‐field views illustrate broader expression in hippocampal pyramidal neurons. Although the entire DG is not uniformly transfected, this localized expression enables functionally meaningful, cell‐specific optogenetic stimulation in the hippocampus. (c) Electrophysiological recordings were acquired from the implanted neural electrode array during remote optogenetic stimulation using 980 nm NIR light. (c1) Raw spike train data recorded from Channel #1 across eight NIR stimulation trials (1 sec per trial) revealed clear increases in neural firing activity during each light‐on period (highlighted in yellow), demonstrating both the successful optogenetic activation and the dual utility of Channel #1 for electroporation‐mediated gene delivery and subsequent neural spike recording. (c2) The corresponding PSTH shows a robust increase in multi‐unit activity (MUA) aligned with the stimulation window, confirming time‐locked neuronal responses to NIR‐driven blue light emission. (c3) Spike sorting analysis distinguished six individual hippocampal neurons contributing to the recorded MUA, supporting the specificity and effectiveness of the UCNP@AuIO‐enabled, fiberless intracranial optogenetic interface in vivo.

To demonstrate the feasibility of remote optogenetic modulation in vivo, we developed and implemented a multifunctional implantable UCNP@3D AuIO modified electrode array that integrates gene delivery, localized electroporation, fiberless NIR‐driven intracranial optogenetic stimulation, and neural recordings within a single platform. These 3D nanostructures served as a high‐surface‐area scaffold that enhanced both biofunctionalization and light transduction, while also improving gene loading efficiency and recording stability.^[^ ^87^, ^88^ ^]^ Following stereotactic implantation into the mouse hippocampal DG region (**Figure** **5**a), an electroporation was applied to facilitate membrane permeabilization and initiate ChR2‐mCherry gene transfection. Fluorescence imaging conducted revealed strong mCherry expression surrounding the implant site (Figure 5b), confirming effective transfection within the DG pyramidal layer. Analysis of both T2‐weighted MR and fluorescence images confirmed the precise targeting of the hippocampal subregion by the tip of the neural electrode array. The pronounced increase in red fluorescence in the electroporated area, without signs of cell apoptosis, indicates that the electrical transfection protocol achieved localized opsin delivery with minimal cytotoxicity.^[^ ^89^, ^90^ ^]^

To validate the functional expression of ChR2, we conducted in vivo extracellular recordings from the implanted site 7 days after electroporation. Upon NIR stimulation, the UCNPs embedded within the GelMA matrix converted the NIR light into locally confined blue luminescence, sufficient to activate ChR2‐expressing neurons in the hippocampal DG region. Neural spike recordings revealed robust, time‐locked discharges corresponding to the onset of each light pulse (Figure 5c1). The peri‐stimulus time histogram (PSTH) aggregated across eight stimulation trials showed a marked increase in MUA spike counts during the light stimulation window (Figure 5c2), demonstrating successful remote activation of opsin‐expressing neurons. Further spike sorting analysis identified six distinct hippocampal neurons that responded reliably to NIR stimulation (Figure 5c3), reinforcing the efficacy of our optoelectronic interface.

The UCNP@3D AuIO‐modified microelectrode array developed in this study presents several significant advantages over existing fiberless or nanoscale photonic neuromodulation systems.^[^ ^44^, ^49^, ^78^, ^91^, ^92^ ^]^ The 3D AuIO nanostructure enhances electrical conductivity and surface area, enabling high‐efficiency loading of non‐viral NT‐PEI‐ChR2 nanocomplexes and maintaining stable electrophysiological recordings.^[^ ^88^ ^]^ In contrast, many prior systems lack such integration and instead rely on dispersed particles^[^ ^49^, ^91^, ^92^ ^]^ or single‐function modules without chronic recording capabilities.

By directly jet‐printing UCNPs encapsulated in GelMA onto the microelectrode surface, our platform achieves highly localized NIR‐to‐blue light conversion. This strategy avoids the use of penetrating optical fibers implanted directly into the brain parenchyma, thereby reducing surgical complexity and minimizing associated tissue damage.^[^ ^76^, ^91^ ^]^ In contrast, some comparator studies^[^ ^44^, ^78^, ^91^, ^92^ ^]^ rely on diffusely distributed photothermal particles or NIR‐absorbing materials, which offer less spatial precision and do not support optogenetic activation of defined neural populations.

A particularly notable outcome of our approach is the ability to achieve deep‐brain activation^[^ ^40^, ^93^, ^94^ ^]^ at a depth of approximately 1.5 mm beneath the skull using 30 mW/mm^2^ of surface‐applied NIR illumination. This performance is enabled by the LSPR‐enhanced upconversion of UCNPs embedded within the 3D AuIO framework, which efficiently transduces near‐infrared light into localized visible excitation for opsin activation.

Importantly, our burst‐mode, low‐duty‐cycle (12%) pulsed stimulation protocol (125 ms pulse width, 4 Hz repetition, 1‐sec window) ensures safe scalp‐skin irradiation at 980 nm. Under these parameters (irradiance = 3.0 W/cm^2^, 4 mm beam diameter), the calculated radiant exposure per pulse (0.375 J/cm^2^) remains well below the ANSI Z136.1/IEC 60825‐1 skin maximum permissible exposure (MPE) of ≈ 2.37 J/cm^2^,^[^ ^95^ ^]^ corresponding to only ≈21% of the accessible emission limit (AEL) for a 3.5 mm limiting aperture. Consequently, the time‐averaged tissue exposure (≈ 1.9 mW over 1 sec) is markedly reduced, minimizing thermal load while preserving a broad safety margin against heating‐induced injury.

These safety considerations were further substantiated by thermal imaging analyses (Figure S12, Supporting Information), which clearly indicated that UCNP@AuIO‐mediated stimulation did not induce any detectable temperature elevation at the probe–tissue interface, in contrast to the visible light‐based micro‐LED system. This observation is consistent with previous reports on UCNP‐mediated deep brain stimulation, where temperature increases of less than 1 °C were recorded under comparable NIR illumination conditions.^[^ ^91^ ^]^

Collectively, these findings validate the thermal safety and spatial confinement advantages of the UCNP@AuIO approach. Unlike micro‐LEDs that directly emit visible light into biological tissue and may cause volumetric heating even at moderate optical powers,^[^ ^96^ ^]^ the present system spatially separates the energy delivery (NIR excitation) from the emission site (electrode), thereby enabling localized activation with minimal photothermal impact. Furthermore, while Jin et al.^[^ ^49^ ^]^ reported an alternative NIR‐induced stimulation mechanism based on UCNP/WO_3‐x_ hybrids that generate localized photocurrents, their method lacks opsin‐targeting specificity and exhibits relatively low conversion efficiency under comparable optical power inputs. In contrast, the LSPR‐coupled upconversion strategy described herein achieves robust yet thermally safe optogenetic modulation by combining efficient deep‐tissue light delivery with full compliance to established laser‐safety standards.

Finally, the multifunctional design of our neural electrode array integrates non‐viral genetic delivery, photonic stimulation, and real‐time electrophysiological recording, which enables closed‐loop modulation of neural circuits with high spatial and temporal resolution.^[^ ^27^, ^97^, ^98^ ^]^ In contrast to prior studies,^[^ ^44^, ^49^, ^91^, ^92^ ^]^ which are largely limited to stimulus‐only or open‐loop systems, our platform provides real‐time feedback and adaptive control, essential for precise and dynamic neural interfacing. To provide a structured comparison of the technical features across these platforms, Table S5 (Supporting Information) shows the key differences between our system and representative studies.^[^ ^44^, ^49^, ^91^, ^92^ ^]^ As summarized in Table S5 (Supporting Information), our platform uniquely integrates gene delivery, NIR‐mediated fiberless intracranial optogenetic stimulation, and closed‐loop neural recording within a compact, biocompatible design. Unlike conventional systems with limited functions or poor targeting specificity, our system enables minimally invasive, chronically stable, and cell‐type‐specific neuromodulation capabilities, making it a strong candidate for next‐generation, minimally invasive neural interfaces.

To further contextualize the functional scope and translational implications of our platform design, we acknowledge and elaborate on the extent of spatial coverage achieved in gene delivery and photostimulation, and its relevance to behavioral and circuit‐level modulation. The current platform was designed to minimize invasiveness while demonstrating localized, fiber‐free optogenetic modulation. In this implementation, electroporation‐driven gene delivery was performed via outermost channels of neural electrode array (Ch#1 and Ch#4, spaced ≈480 µm along the DV axis). This allowed for ChR2‐mCherry expression adjacent to the electrode tract, with partial transfection observed in neighboring CA1 regions. Concurrently, UCNPs printed onto Ch#2 and Ch#3 generated blue light over a ≈180 µm region, providing spatially confined photostimulation. While this configuration does not fully transfect or stimulate the entire DG volume (≈5.6 mm^3[^ ^99^ ^]^), it was purposefully engineered for focal activation to evaluate proof‐of‐concept functionality. Traditional optogenetic approaches often require high‐volume viral injections to ensure whole‐nucleus coverage. However, such strategies may cause tissue compression, cytotoxicity, or off‐target expression, particularly when multiple injection sites are needed.^[^ ^38^, ^100^, ^101^, ^102^, ^103^, ^104^ ^]^

Importantly, emerging evidence in systems neuroscience suggests that activation of small neural populations^[^ ^105^, ^106^ ^]^ or even single neurons^[^ ^107^, ^108^, ^109^, ^110^ ^]^ can significantly impact perception and behavior. These findings highlight that behavioral modulation does not universally require full‐nucleus coverage, especially when targeting functionally specific ensembles. Our current probe design, although limited in stimulation volume, establishes a framework for such selective, spatially aligned activation. Future extensions such as multi‐site electroporation, volumetric probe geometries, or hybrid viral‐electroporation strategies can enable broader expression while retaining spatial precision and biosafety.

## 3 Conclusion

In this study, we developed a multifunctional neural interface that unifies non‐viral delivery of AAV‐derived plasmid, fiberless NIR‐driven intracranial optogenetic stimulation, and real‐time neural recording into a single implantable platform. By integrating a 3D AuIO microelectrode with embedded UCNPs and localized gene nanocomplexes (NT‐PEI‐ChR2), the system enables precise, spatially targeted, and minimally invasive neuromodulation in deep brain regions. The LSPR‐enhanced UCNPs facilitate efficient NIR‐to‐visible conversion at reduced power levels, while the porous AuIO architecture provides a stable scaffold for both electroporation and signal acquisition.

This all‐in‐one interface eliminates the need for intracranially implanted optical fibers, thereby reducing surgical invasiveness and tissue damage associated with conventional optogenetic systems. More importantly, its ability to simultaneously deliver optical stimulation and record electrophysiological feedback provides a foundation for chronic, cell‐specific, and closed‐loop‐capable modulation of deep neural circuits. Such an integrated system represents a critical step toward next‐generation brain–machine interfaces and personalized neuromodulation therapies for neurological and psychiatric disorders.

Although the present study did not implement real‐time feedback control, the system architecture was intentionally designed to support such functionality in future applications. Achieving real‐time closed‐loop neuromodulation will require the use of disease‐specific models and the identification of reliable electrophysiological biomarkers, both of which remain key objectives for our subsequent investigations.

## 4 Experimental Section

### Chemicals and Reagents

Oleic acid (90%; CAS. No. 112‐80‐1), 1‐Octadecene (90%; CAS. No. 112‐88‐9), Yb (CH_3_CO_2_) _3_·4H_2_O (99.9% trace metals basis; CAS. No. 15280‐58‐7), Tm (CH_3_CO_2_)_3_·× H_2_O (99.9% trace metals basis; CAS. No. 207738‐11‐2), Y(CH_3_CO_2_)_3_·× H_2_O (99.9% trace metals basis; CAS. No. 304675‐69‐2), NH_4_F (ACS reagent, ≥ 98%; CAS. No. 12125‐01‐8), HAuCl_4_ (CAS. No. 16903‐35‐8), 25k‐branched polyethylenimine (PEI; CAS. No. 9002‐98‐6), Neurotensin (NT; CAS. No. 39379‐15‐2), Dimethyl Sulfoxide (DMSO; CAS. No. 67‐68‐5), Phosphate buffered saline (PBS, pH = 7), Laminin (CAS. No. 114956‐81‐9), Gelatin (CAS. No. 9000‐70‐8). All purchased from Sigma‐Aldrich, Munich, Germany. Indium tin oxide (ITO, Uni‐onward Corp., Taipei, Taiwan). SYLGARD 184 Silicone Elastomer Kit (PDMS, GOALBio, Taipei, Taiwan). Agarose (CAS. No. 9012‐36‐6, FocusBio, Philadelphia, USA), Plasmid DNA (pcDNA3.1/hChR2(H134R)‐mCherry, Addgene, Massachusetts, United States), *N*‐Succinimidyl 3‐(2‐pyridyldithio) propionate (SPDP, CAS. No. 141‐78‐6; Thermo Fisher Scientific, Massachusetts, USA), 2‐Iminothiolane‐HCl (Traut's reagent, CAS. No. 4781‐83‐3; Thermo Fisher Scientific, Massachusetts, USA), Quant‐iTTM Picogreen dsDNA Reagent and Kits (Invirtogen, Massachusetts, USA).

### Synthesis and Characteristics of NT‐PEI‐ChR2

**Figure 6:**
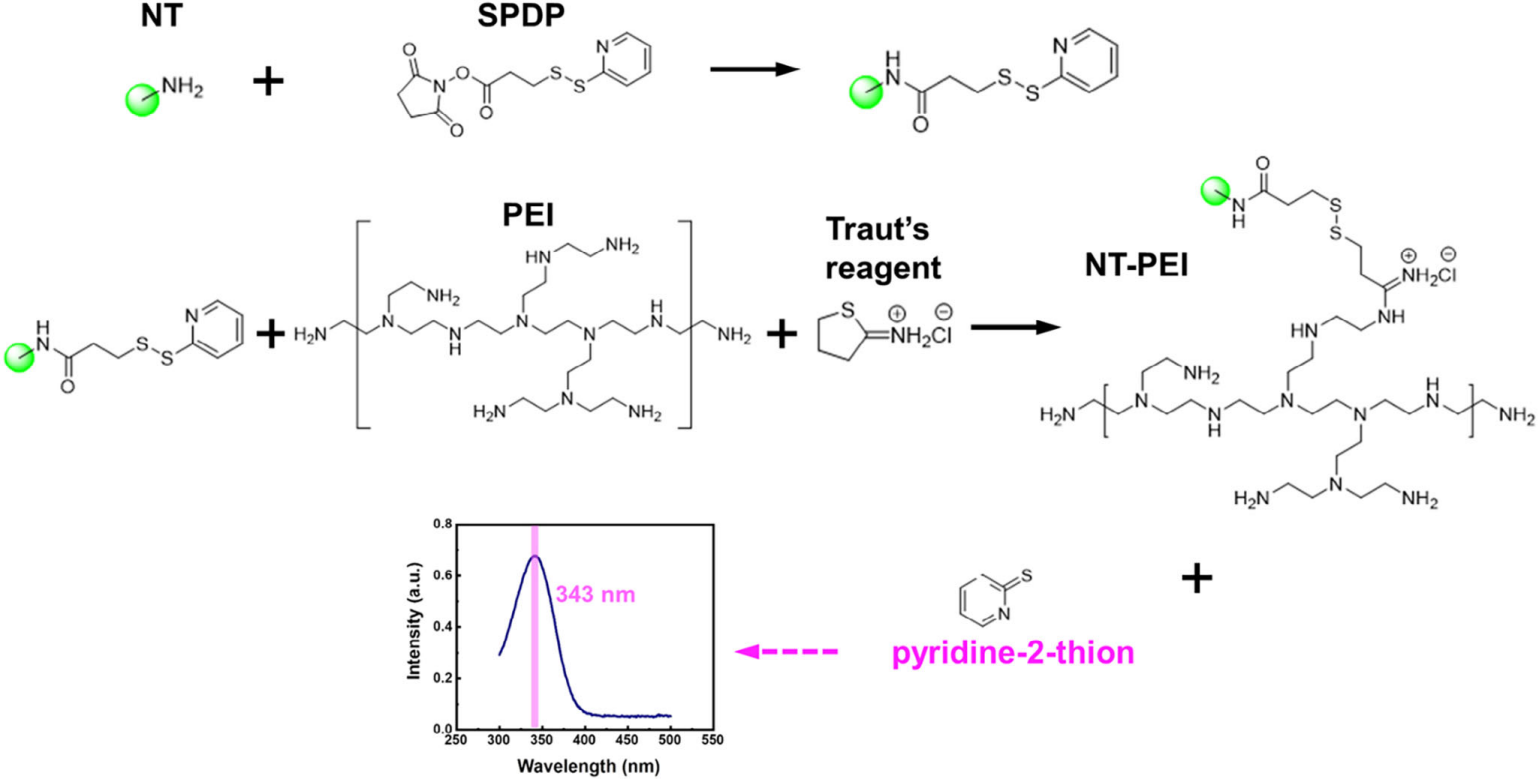
Schematic of NT‐PEI‐ChR2 synthesis. NT was first reacted with SPDP to introduce a pyridyldisulfide group. The modified NT was then conjugated to PEI using Traut's reagent, forming NT‐PEI, confirmed by pyridine‐2‐thione release (absorbance at 343 nm). NT‐PEI was then complexed with ChR2‐plasmid DNA.

The chemical synthesis route^[^ ^17^ ^]^ of the NT‐PEI‐ChR2 nanocomplex is illustrated in **Figure** **6**. Briefly, neurotensin (NT, 10 mg/mL) was first reacted with succinimidyl 3‐(2‐pyridyldithio) propionate (SPDP, 22 nM) to introduce a pyridyldisulfide functional group, and the mixture was stirred at room temperature for 30 minutes. Subsequently, polyethylenimine (PEI, 2 mg/mL) and Traut's reagent (100 nM) were added to the reaction solution and allowed to react under gentle stirring for 8 h to facilitate conjugation.

Successful formation of NT‐PEI was confirmed by monitoring the release of pyridine‐2‐thione, which exhibited a characteristic absorbance peak at 343 nm, indicating effective displacement. The NT‐PEI conjugate was then mixed with ChR2 plasmid DNA and incubated with gentle stirring for min to form the NT‐PEI‐ChR2 nanocomplex. The final complex was stored at 4 °C until use.

The zeta potentials of free ChR2 plasmid DNA, PEI‐ChR2, and NT‐PEI‐ChR2 complexes were measured using a Zetasizer Nano ZS90 instrument (Malvern Instruments Ltd., Worcestershire, UK). For each measurement, the samples were diluted in deionized water to a final concentration of 10 µg/mL and equilibrated at 25 °C prior to analysis.

### Controlled Release Experiments for NT‐PEI‐ChR2 from Planar and 3D AuIO Electrodes—Fabrication of 3D AuIO Electrodes on the ITO Substrate

The 3D nanostructured AuIO‐modified microelectrodes were fabricated by first synthesizing polystyrene (PS) microspheres with an average diameter of 700 nm via a soap‐free emulsion polymerization process,^[^ ^111^ ^]^ followed by their homogenous suspension in an anhydrous ethanol to form a 1 wt% colloidal solution with the pH value adjusted to 9 with the addition of a minute amount of NH_4_OH. Subsequently, an electrophoretic deposition was employed in which an electric field of 8 V/cm was imposed for 12 min to direct the PS microspheres to form a close‐packed colloidal crystal on the Au working electrode (dimension 1 cm^2^ on ITO‐coated glass substrate). A stainless‐steel plate (25 cm^2^) was used as the counter electrode. After being withdrawn from the PS suspension, the PS colloidal crystal was dried in an oven at 60 °C for 3 days to stabilize the microstructure.^[^ ^112^, ^113^ ^]^ A sacrificial nickel (Ni) layer was then electrodeposited into the interstitial voids of the PS template using 0.5 M NiSO_4_ electrolyte at a constant current of 40 mA for 1 h. Subsequently, gold was backfilled into the upper layers via electrodeposition from 5 mM HAuCl_4_ at 0.8 V for 45 min. Following deposition, the PS microsphere template was removed by immersing the sample in toluene at 60 °C, followed by a thermal‐assisted water bath treatment, resulting in the formation of a porous 3D AuIO film atop the underlying Ni layer. The Ni layer was subsequently subjected to selective etching to yield the AuIO‐modified microelectrodes. For reference, the fabrication procedure for planar gold nanofilm electrodes is described in detail in Note S5 (Supporting Information).

The surface of both 3D AuIO and planar Au electrodes was pretreated with oxygen plasma for 5 min to improve hydrophilicity and promote uniform loading. Subsequently, 60 µL of NT‐PEI‐ChR2 solution (2 µg/mL) was pipetted onto the surface of 3D AuIO and planar Au electrode and allowed to adsorb into the porous matrix (in the case of AuIO) for 30 min at room temperature. The electrodes were then gently rinsed to remove unbound complexes and transferred into individual wells of a 24‐well plate, each containing 0.5 mL PBS for release testing.

### Electrically‐Controlled Release From the Fabricated Electrodes

To investigate the effects of electrical stimulation and electrode geometry on gene complex release behavior, we applied controlled biphasic electrical pulses using an isolated pulse stimulator (Model 2100, AM‐Systems, USA). Three current intensities (50, 100, and 200 µA) were tested, with fixed parameters: 1 Hz frequency and 250 ms pulse width. Cumulative release was monitored over a 120‐min period. Additional stepwise release tests were conducted, in which stimulation was applied for 5 min, followed by 15‐min resting intervals, to simulate programmable release conditions. Release quantification was performed by labeling the released DNA with PicoGreen dsDNA reagent (Thermo Fisher Scientific, USA), and fluorescence intensity was measured using a microplate reader (CLARIOstar, BMG Labtech, Germany) at excitation/emission wavelengths of 480/520 nm. The comparative experiments included both 3D AuIO and planar Au electrodes under identical loading and stimulation conditions, with or without electrical actuation. These experiments enabled quantitative assessment of geometry‐dependent and stimulation‐dependent release kinetics for NT‐PEI‐ChR2 complexes.

### Localized Electroporation‐Mediated Non‐Viral Gene Delivery via 3D AuIO‐Modified MEA—Adaptation of 3D AuIO Nanostructure Fabrication for MEA and Neural Electrode Array

To enable site‐specific gene delivery via localized electroporation, the 3D AuIO nanostructures described in Section 4.3.1 were adapted and directly fabricated onto selected microelectrodes of our lab‐designed MEA chip. Briefly, the same electrophoretic deposition protocol was applied to assemble PS microspheres (diameter ≈800 nm) onto individually addressable MEA microelectrode sites (diameter: 30 µm) under an electric field of 8 V/cm for 10 min. After drying at 60 °C for 48 h, the Ni sacrificial layer and subsequent Au backfilling were performed using the same electrochemical parameters as described in Section 4.3.1, with minor adjustments to deposition time (Ni: 1 h at 40 mA; Au: 30 min at 0.8 V) to accommodate the reduced surface area. The PS template was then removed via toluene immersion and water rinsing, yielding a highly porous AuIO morphology precisely integrated onto the microelectrode surfaces. This approach was also successfully applied to neural electrode arrays featuring smaller microelectrode diameters (≈15 µm), demonstrating the versatility and scalability of the fabrication method across electrode platforms while preserving microscale addressability required for localized electroporation and optical modulation.

### Preparation of 3D AuIO‐Modified MEA Loaded With Non‐Viral Gene Vectors

Two types of gene complexes were prepared: NT‐modified non‐viral opsin vector (NT‐PEI‐ChR2‐mCherry) and unmodified vector (PEI‐ChR2‐mCherry). Each complex was diluted to 2 µg/mL in PBS and pipetted (2 µL per site) onto paired microelectrodes bearing the 3D AuIO nanostructures. Prior to loading, microelectrodes of MEA platform were oxygen plasma‐treated for 5 min to enhance hydrophilicity. The loading was allowed to proceed at room temperature for 30 min, ensuring sufficient infiltration into the porous matrix. The remaining solution was gently removed and replaced with differentiation medium.

A 3‐cm acrylic ring was affixed around the MEA to create a culture reservoir. MEAs were sterilized by UV exposure and coated with laminin (10 µg/mL) to promote cell adhesion. PC‐12 cells (1.5 × 10^5^ cells per chip) were seeded and incubated for 24 h in RPMI‐1640 medium. The following day, the medium was replaced with differentiation medium containing nerve growth factor (NGF, 100 ng/mL). After 4 days of differentiation, gene complexes were introduced, and electroporation was performed using a paired microelectrode configuration to define localized transfection regions.

### In Vitro Investigation on Electroporation on the MEA Platform

Electroporation was performed using a constant biphasic square current of 100 µA at 120 Hz with MODEL 2100 Isolated pulse stimulator (A‐M Systems, Sequim, WA, USA), delivered through the selected microelectrode pair. The electroporation protocol (E^+^) consisted of three cycles, each comprising 5 min of electroporation followed by 10 min of rest. Control groups (E^−^) underwent identical handling but without electroporation. After transfection, cells were maintained at 37 °C in a 5% CO_2_ incubator and observed under fluorescence microscopy after 24–48 h.

To quantify gene transfection efficiency, cells were harvested 48 h post‐transfection. Cells were washed twice with PBS, centrifuged, and resuspended in PBS containing 2% FBS. Flow cytometry was performed using a BD FACSCanto II cytometer (BD Biosciences, Franklin Lakes, NJ, USA). mCherry fluorescence was detected in the PerCP‐Cy5.5 channel (excitation: 488 nm; emission: 695 ± 20 nm). A minimum of 10 000 events per sample were recorded, and appropriate compensation and gating were applied using untransfected controls. Data analysis was performed with FlowJo (version 10.8; FlowJo LLC, Ashland, OR, USA), and transfection efficiency was reported as the percentage of mCherry‐positive cells. All measurements were performed in biological triplicates (*n* = 3).

### Integration of UCNPs into Microelectrodes by Aerosol‐Jet Printing—Synthesis of UCNPs and Preparation UCNP Ink

NaYF_4_: Yb^3+^, Tm^3+^ UCNPs were prepared by using our previously reported method.^[^ ^48^ ^]^ Briefly illustrates, 50 mL two‐neck bottle containing the mixture of R(CH_3_CO_2_)_3_ (R = Y, Yb, Tm), 8 mL oleic acid, and 12 mL 1‐octadecene, then heating to 150 °C to evaporate the containing water. Secondly, methanol‐based solution containing 2 mL NaOH (1 mM) and 7.9 mL NH_4_F (1.2 mM) were added to above solution. After evaporating methanol, kept the solution under nitrogen atmosphere and heating to 310 °C. Collecting UCNPs by centrifuge at 8000 rpm for 10 min after cooling to room temperature.

To achieve precise 3D printing of UCNPs onto the AuIO sites of microelectrodes, a combination of chemical modification and Aerosol Jet printing was employed. Initially, the hydrophobic UCNPs dispersed in an oil phase were rendered hydrophilic by removing their oleic acid ligands through an acid‐induced ligand stripping method. Specifically, a mixture solution of 2 M HCl and ethanol (1:1, *v/v*) was added to the oleic acid‐dissolved UCNPs and stirred at 60 °C for 5 h. Following the ligand removal, the resulting aqueous‐phase UCNPs were dispersed into a pre‐warmed mixture containing 3% GelMA (gelatin methacryloyl) and a crosslinker 0.5 wt% 2‐Hydroxy‐4‐(2′‐hydroxyethoxy)‐2‐methylpropiophenone (Irgacure 2959, MilliporeSigma, Burlington, MA, USA) at 45 °C to formulate the printable ink.

### 4.1 Printing of UCNP Ink on of MEA and Neural Electrode Arrays

The prepared UCNP ink was then printed onto the 3D nanostructured AuIO‐modified microelectrodes of the MEA chip (microelectrode diameter: 30 µm) or the neural electrode array (microelectrode diameter: 15 µm) using an aerosol jet printing method (HD2, Optomec Inc., Albuquerque, NM, USA). In this method, the ink was first atomized using ultrasonic waves (atomizer) and then directed toward the substrate with a focused sheath gas flow. The printing nozzle traversed a precisely defined circular trajectory with a diameter of less than 30 µm, enabling accurate deposition of the ink onto designated AuIO‐modified microelectrodes. After printing, the deposited hydrogel‐based ink was photo‐crosslinked and fixed in place by exposure to UV light, ensuring the stable integration of the UCNPs onto the microelectrode structures. Note S11 (Supporting Information) summarizes the aerosol jet printing process conditions for GelMA‐UCNP deposition (optical transducer layer), incorporating the machine specifications.

### Evaluation of Upconversion Efficiency of UCNP Based on AuIO‐Modified Microelectrodes

To quantitatively evaluate the UCL behavior and emission efficiency of UCNP‐modified microelectrode platforms, we established a custom optical measurement system, as depicted in Figure S11 (Supporting Information). The setup employs a Y‐type bifurcated optical fiber, where one branch connects to a continuous‐wave 980 nm NIR laser diode (Model: MDL‐E‐980, New Industries Optoelectronics Tech. Co., Ltd., Changchun, China), providing excitation light, and the other branch routes the emitted signal to a CCD‐coupled spectrometer (Model: CCS200, Thorlabs Inc., Newton, NJ, USA) for spectral analysis. The shared end of the bifurcated fiber is coupled to a 50 × objective lens mounted on a micro‐Raman microscope, allowing precise focusing and spatially resolved measurement on the microelectrode surface of the electrode array. This dual‐function fiber enables simultaneous NIR excitation and emission signal collection from the same optical path. The laser output is controlled via a digital driver (Model: LD10CHA, New Industries Optoelectronics Tech. Co., Ltd., Changchun, China), and an inline fiber‐coupled photodiode sensor (Model: S151C, Thorlabs Inc., Newton, NJ, USA), connected to a power meter (Model: PM100D, Thorlabs Inc., Newton, NJ, USA), monitors the incident power to ensure consistent excitation conditions. This configuration facilitates accurate and localized quantification of UCNP emission intensity across microelectrode sites, with or without 3D AuIO nanostructures.

To estimate the power density (*E*_475_, in mW/mm^2^) of NIR‐to‐blue emission at 475 nm for different microelectrode configurations (Au, UCNP@Au, and UCNP@AuIO) of the electrode array, the integrated spectral intensity at the 475 nm emission band (*I*_475_) was normalized against the total integrated intensity over the 400–1100 nm range to minimize experimental variability in excitation power or detection efficiency. The resulting blue light irradiance was then calculated using the following equation:

(1) $$E_{475}=\left(\frac{I_{475}}{I_{\mathrm{total}}}\right)\cdot E_{\mathrm{total}}$$

where *I*_475_ denotes the integrated spectral intensity within the 450–500 nm range, corresponding to the blue upconversion emission centered at 475 nm, and *I_total_* is the integrated spectral intensity over the 400–1100 nm range acquired from the CCD‐coupled spectrometer (Model: CCS200, Thorlabs Inc., Newton, NJ, USA), serving as an internal reference. The total output power was first measured using a calibrated fiber‐coupled photodiode sensor (Model: S151C, Thorlabs) connected to a power meter (Model: PM100D). The power meter reading represents the integrated optical power between 400 and 1100 nm, which matches the spectral detection window of our system. This value was then divided by the active area of the sensor (3.6 mm × 3.6 mm = 12.96 mm^2^) to calculate the total irradiance (*E_total_*).

### Animals and Surgery Group

Experiments were conducted with male wild‐type (C57BL/6J) mice (postnatal day 60–82, purchased from BioLASCO Taiwan Co., Ltd., Taipei, Taiwan). Each animal represents an individual experiment, performed once. In total, 10 mice were used for this study. All experiments were approved by the Institutional Animal Care and Use Committee at Taipei Medical University (IACUC Approval No: LAC‐2020‐0210). Every effort was taken to minimize the number of animals used and animal suffering. All mice were housed at a controlled temperature (25 °C) in a 12 h/12 h day/night cycle with free access to rodent food and water. To evaluate the feasibility and functional transduction capabilities of remote optogenetic stimulation using our UCNP‐3D AuIO interface, we first conducted ex vivo proof‐of‐concept experiments (*n* = 5). In these experiments, UCNPs were integrated onto a 3D nanostructured AuIO‐modified MEA chip, enabling localized conversion of NIR light into visible blue emission. This setup allowed for wireless, spatially resolved activation of ChR2‐expressing neurons in hippocampal slices. To further demonstrate minimally invasive, wirelessly controlled optogenetic neuromodulation and neural recording in deep‐brain regions, we performed in vivo proof‐of‐concept experiments (*n* = 5) using a multifunctional neural electrode array integrated with UCNPs. This device combined electroporation‐mediated gene delivery, NIR‐responsive optogenetic stimulation, and electrophysiological recording, thereby validating the technological feasibility and functional performance of a wirelessly addressable optogenetic interface.

### Ex vivo Experiments

To establish a robust proof of concept for remote optogenetic neuromodulation on hippocampal slices, we conducted an ex vivo electrophysiological study utilizing our laboratory‐designed MEA chip^[^ ^65^ ^]^ integrated with UCNPs immobilized on a 3D AuIO substrate. This UCNP‐3D AuIO‐modified MEA was specifically designed to achieve localized transduction of NIR light into visible blue emission, thereby enabling wireless, spatially resolved activation of opsin‐expressing neurons.

### Stereotaxic Viral Injection For Ex vivo Experiment

Five mice were administered with anesthesia with isoflurane (4% induction and 1.5–2% maintenance in air and oxygen mixed gas) and placed in a stereotaxic frame with a mouse adaptor (Item Nos. 51 600 and 51 625, Stoelting Co., Wood Dale, IL, USA). Body temperature was maintained at 37 °C by a controlled heating pad. For ex vivo slice experiments, AAV9‐CaMKIIa‐hChR2‐mCherry (the original titer 3 × 10^13^ copies/mL, diluted to 1/10 in physiological saline; Catalog # 26975‐AAV9, Addgene, Cambridge, MA, USA) were injected unilaterally in the DG region of hippocampus (coordinates relative to bregma: −2.1 mm AP, +1.5 mm ML, and ‐1.5 mm DV). Virus was injected at a volume of 250–300 nL at a flow rate of 20 nL/min using a 30‐guage needle affixed to a 10 µL Hamilton syringe (Model 701 N, Hamilton Company, Reno, NV, USA) and a syringe pump (KDS 310, KD Scientific Inc, New Hope, PA, USA). After viral injection, the needle was left in place for at least 10 min before withdrawal.

### Hippocampal Slice Preparation

Mice more than 2 weeks after viral injection were decapitated under deep isoflurane anesthesia and was rapidly removed and placed in cold (4 °C) sucrose‐based cutting solution saturated with 95% O_2_ and 5% CO_2_ containing the following (in mM): 87 NaCl, 2.5 KCl, 1.25 NaH_2_PO_4_, 7 MgSO_4_, 0.5 CaCl_2_, 25 NaHCO_3_, 25 glucose. Coronal slices of 400‐mm thickness were prepared with a Leica VT1200S vibratome (Leica Microsystems, Wetzlar, Germany) and transferred to artificial cerebrospinal fluid (ACSF; saturated with 95% O_2_ and 5% CO_2_) containing the following (in mM): 124 NaCl, 2.5 KCl, 1.25 NaH_2_PO_4_, 4 MgSO_4_, 4 CaCl_2_, 26 NaHCO_3_, and 10 glucose. After incubation of the slices at 36 °C for 60 min, slices were shifted to at room temperature for at least 90 min and until slice electrophysiology experiments.

### Ex Vivo Slice Electrophysiological Recordings and Remote NIR Optogenetic Photostimulation

For experiments, individual slices were transferred to our UCNP‐3D AuIO‐modified MEA platform and then were continuously perfused with ACSF saturated with 95% O_2_ and 5% CO_2_ containing the following (in mM): 125 NaCl, 25 NaHCO_3_, 1.25 NaH_2_PO_4_, 2.5 KCl, 25 glucose, 2 CaCl_2_, and 1 MgCl_2_. The ChR2‐mCherry expression region within the hippocampal slice was identified using epifluorescence imaging. This region was then visually aligned and positioned over the UCNP‐3D AuIO‐modified microelectrode using an infrared Dodt gradient contrast optical system (DM6000 CFS, Leica Microsystems, Wetzlar, Germany), in preparation for subsequent NIR optogenetic photostimulation and slice electrophysiological experiments.

All slice electrophysiological experiments were performed using our laboratory's custom‐designed MEA recording system, originally described by Chang et al. (2012).^[^ ^65^ ^]^ Hippocampal slices were then transferred to our MEA recording chamber, which contains 59 UCNP‐3D AuIO‐modified microelectrodes (30 µm diameter, 200 µm inter‐microelectrode pitch) and one reference electrode microfabricated on a glass substrate and interfaced to a low‐cost headstage and acquisition module as detailed in Chang et al. (2012).^[^ ^65^ ^]^

A 980 nm NIR laser (K980F11CA‐10.00W, BWT Beijing Ltd., China), coupled to a collimated optical fiber, was vertically positioned 30 mm above the tissue surface. The beam was collimated to a ≈3 mm diameter spot, spatially matching the ≈60‐microelectrode region of the MEA recording chamber to ensure uniform optical coverage. The MEA chamber incorporated a transparent optical window, enabling direct illumination without physical or optical interference. The 30 mm standoff distance was optimized to maximize photon delivery to the UCNP layer while minimizing mechanical perturbation and local thermal effects. The laser power at the tissue interface was calibrated using a power meter (PM100D with S151C sensor, Thorlabs Inc., Newton, NJ, USA) to achieve an output intensity of 25–30 mW/mm^2^, which falls within the effective range for ChR2 activation. A burst‐mode protocol was triggered and synchronized to the MEA acquisition system via TTL pulses generated by the laboratory's control interface. A burst‐mode photostimulation protocol was employed, consisting of ten trains of four light pulses (125 ms per pulse), with a 250 ms interstimulus interval (ISI) and a 2‐sec inter‐train interval (ITI), designed to emulate physiologically relevant neural activation patterns.

Extracellular potentials from all 60 channels were simultaneously sampled at 25 kHz per channel using a 16‐bit onboard A/D converter. Signals were online band‐pass filtered between 300 and 3000 Hz to isolate multiunit spike activity and digitally notch‐filtered at 60 Hz to suppress line noise, following our previously established configuration.^[^ ^65^ ^]^ Data were streamed in real time to custom‐designed acquisition software for visualization and storage.

Offline, raw traces were processed to detect neural spikes using a threshold crossing method, with thresholds set at 5× the standard deviation of the bandpass‐filtered signal. Detected spike waveforms (1.5 ms window) were subjected to principal component analysis (PCA), and single units were isolated via k‐means clustering in PCA space.^[^ ^25^ ^]^ Units were classified as single neurons based on consistent waveform morphology and clear refractory periods in their autocorrelograms.^[^ ^114^ ^]^ For quantification, multiunit activity (MUA) spike counts were computed within 1‐sec light‐ON epochs across trials to assess photostimulation‐evoked responses.

### In Vivo Experiments

For in vivo studies, the plasmid pAAV9‐CaMKIIa‐hChR2‐mCherry (Addgene plasmid #26 975, Cambridge, MA, USA), an AAV‐derived construct, was selected for non‐viral NT‐PEI‐mediated delivery. Importantly, this plasmid was chosen to ensure experimental consistency with the ex vivo validation study described in Section 4.8, where AAV9‐CaMKIIa‐hChR2‐mCherry viral vectors were stereotaxically injected into the DG of the mouse hippocampus. By maintaining the same genetic construct across both viral (ex vivo) and non‐viral (in vivo) delivery experiments, we ensured that any observed differences in opsin expression or neural response could be attributed to the delivery modality, rather than variability in promoter strength, genetic codon usage, or construct configuration. Notably, no viral packaging or infection steps were involved in the in vivo non‐viral approach; the plasmid backbone was simply retained for standardization and compatibility.

Prior to neural implantation, the 3D AuIO‐modified neural electrode array underwent a two‐step, spatially resolved functionalization process using an aerosol jet printing system to ensure high‐precision deposition of both the optical and genetic components: (1) GelMA‐encapsulated UCNPs were printed onto Channel #2 and Channel #3 using aerosol jet printing (see the aerosol jet printing parameters for GelMA‐UCNP deposition in Table S3, Supporting Information), enabling conformal and uniform coatings over the 3D porous microelectrodes. Following deposition, the UCNP‐GelMA ink was UV crosslinked in situ to stabilize the coating and establish robust adhesion for long‐term in vivo optical transduction (Figure S15, Supporting Information). In the subsequent step, (2) PEI–NT–ChR2‐mCherry nanocomplexes were aerosol jet‐printed onto Channel #1 and Channel #4 (see the aerosol jet printing parameters for NT‐PEI‐ChR2 gene nanocomplex deposition in Table S4, Supporting Information), forming discrete, micrometer‐scale patterns. This spatially selective deposition ensured focal gene delivery localized to predefined electroporation sites. The aerosol jet printing system enabled non‐contact deposition with high fidelity, critical for preserving the underlying electrode structure and ensuring effective hybrid integration of optical and genetic modules.

### Animal Surgery and Electroporation‐Mediated Gene In Vivo

All animal experiments were performed using five healthy male mice (*n* = 5). Mice were anesthetized with isoflurane (4% induction, 2% maintenance; Sigma–Aldrich, USA) and mounted in a stereotaxic frame (Stoelting Co., Wood Dale, IL, USA) atop a 37 °C heated platform. A unilateral craniotomy (≈0.5 mm diameter) was performed to access the right hippocampal DG. A custom‐fabricated 16‐channel neural electrode array,^[^ ^17^, ^66^ ^]^ comprising 15 µm diameter Au microelectrodes arranged at 150 µm pitch and modified with 3D AuIO nanostructures, was implanted using standard stereotaxic coordinates: AP −2.0 mm, ML +1.5 mm, DV −1.5 mm.

After securing the array to the skull with stainless steel screws and dental acrylic, localized electroporation was performed using Channel #1 (anode) and Channel #4 (cathode) via an isolated pulse stimulator (Model 2100, AM‐Systems, Carlsborg, WA, USA). The stimulation protocol followed our previously validated parameters for safe and efficient non‐viral gene transfection in brain tissue.^[^ ^17^ ^]^ Post‐electroporation, animals were monitored and allowed to recover for seven days prior to electrophysiological recordings to assess in vivo ChR2 expression and optogenetic response upon NIR stimulation.

### In Vivo Optogenetic Stimulation and Electrophysiological recording

The implanted mice were anesthetized via intraperitoneal injection of a ketamine cocktail (70 mg/kg ketamine, 10 mg/kg xylazine) and positioned in a stereotaxic frame for combined in vivo optogenetic stimulation and electrophysiological recording. The electrode array implanted in the hippocampal DG was connected to the headstage (RHD2132, Intan Technologies, Los Angeles, CA, USA) and interfaced with the Open Ephys acquisition system, sampling at 30 kHz.^[^ ^115^ ^]^ For in vivo optogenetic stimulation, a continuous‐wave 980 nm laser (model: K980F11CA‐10.00W, BWT Beijing Ltd., China) was mounted above the stereotactic platform and coupled to a collimated optical fiber. The fiber tip was positioned 30 mm above the skull surface to deliver vertically incident illumination through the skull, resulting in an approximately 4 mm beam diameter at the skull surface. The calibrated NIR power density at the skull surface was set to 30 mW/mm^2^, measured using a Thorlabs PM100D power meter and S151C photodetector. A burst‐mode photostimulation protocol was employed, consisting of ten trains of four light pulses (125 ms per pulse), with a 250 ms interstimulus interval (ISI) and a 2‐sec ITI. Each stimulation epoch was precisely synchronized to the electrophysiological recording system via TTL pulses triggered concurrently with laser onset.

To evaluate neural responses elicited by photostimulation, electrophysiological signals were continuously recorded during each stimulation trial. These recordings enabled both real‐time visualization of stimulus‐evoked neuronal activity and subsequent offline analysis of spike characteristics. Spike detection was performed using a threshold set at four times the standard deviation of MUAs, extracted from raw signals bandpass‐filtered between 300 and 6000 Hz. PCA was then applied for feature extraction, followed by K‐means clustering to isolate well‐defined single‐unit activities. This spike sorting methodology was consistent with that described previously for the Ex vivo *experiments* (Section 4.8.3), allowing comparative assessment across experimental conditions.

### Immunohistochemistry (IHC) Staining

At the conclusion of the experiment, a small electrolytic lesion was created by delivering an anodal current (30 µA for 10 s) through the deepest microelectrode of the laboratory‐designed neural electrode array, using an isolated pulse stimulator (Model 2100, A‐M Systems, Carlsborg, WA, USA). This lesion served to mark the recording site within the hippocampal DG. Following lesioning, animals were euthanized via an overdose of anesthetic and transcardially perfused with 4% paraformaldehyde in 0.1 M phosphate‐buffered saline (PBS, pH 7.4) at a flow rate of 50 mL/min. The brains were then carefully extracted from the skulls and post‐fixed in a solution of 4% paraformaldehyde with 30% sucrose at 4 °C for 24 h. Subsequently, coronal brain sections were cut at a thickness of 30 µm using a freezing microtome (CM1800, Leica, Berlin, Germany).

Immunohistochemical analysis was conducted to examine ChR2–mCherry expression in fluorescently labeled brain tissue sections following electrically induced in vivo gene transfection. Consecutive brain sections (30  µm thickness) were washed in Tris‐buffered saline (TBS) three times for 5 min each. Blocking was performed with a solution containing 3% normal goat serum and 0.3% Triton‐X in 1× TBS for 30 min at room temperature. Sections were then incubated at 4 °C for 24 h with a rabbit polyclonal anti‐mCherry primary antibody (Cat# ab167453; Abcam, Cambridge, UK), diluted between 1:1000 and 1:5000 in 0.3% Triton‐X in 1 × TBS. After incubation, sections were washed in TBS (3 × 5 min), mounted onto Superfrost Plus microscope slides (Cat# 12‐550‐15; Fisher Scientific International, Inc., PA, Pittsburgh, USA), and coverslipped using DAPI Fluoromount‐G (Cat# 0100‐20; SouthernBiotech, Birmingham, AL, USA).

Fluorescence imaging was performed using a Zeiss LSM 880 confocal laser scanning microscope (Carl Zeiss, Oberkochen, Germany) operated with Zen 2012 SP2 software (v11.0, Carl Zeiss, Oberkochen, Germany). Z‐stack images were acquired using a C‐APOCHROMAT 40×/1.2W objective lens and appropriate laser lines, including a blue diode (405 nm) for DAPI and a helium‐neon (HeNe; 612 nm) laser for mCherry. The hippocampal DG, along with the corresponding implant tracts and electrolytic lesion, were segmented and identified in both T2‐weighted MR and fluorescence images. These were referenced against the Mouse Brain Common Coordinate Framework (CCFv3)^[^ ^116^ ^]^ and the anatomical mouse brain atlas,^[^ ^117^ ^]^ respectively, to verify the anatomical localization of ChR2–mCherry expression within the hippocampal DG and the precise position of the tip of neural electrode array.

## Conflict of Interest

The authors declare no conflict of interest.

## Supporting information



Supporting Information

Supporting Information

Supporting Information

## Data Availability

The data that support the findings of this study are available in the supplementary material of this article.
