# Supplementary material for: A Closed‐Loop‐Capable Neural Interface Platform for Deep Brain Modulation via Integrated Non‐Viral Gene Delivery, NIR Optogenetics, and Electrophysiological Recording
Source: Adv Sci (Weinh). 2025 Nov 19;13(7):e15060. doi: 10.1002/advs.202515060 (PMC12866705; doi:10.1002/advs.202515060)
Supplement: Supplementary file 1 — Supporting Information [file ADVS-13-e15060-s002.docx]

***Supplementary Materials***

**A Closed-Loop****-Capable Neural Interface Platform for Deep Brain Modulation via Integrated Non-Viral Gene Delivery, NIR Optogenetics, and Electrophysiological Recording**

Chao-Yi Chu^1†^, Zih-Huei Chen^7†^, Chun-Wei Liang^1†^, Pu-Wei Wu^1^, Wei-Qing Guo^1^, Bo-Wei Chen^2^, Chih-Chia Huang^3,4^, Ssu-Ju Li^2^, Ching-Wen Chang^2^, Yao-Wen Liang^2^, Shun-An Kan^5^, Yu-Chun Lo^6^, Wei-Chen Huang^7, *^, You-Yin Chen^2,6, *^, and San-Yuan Chen^1,8,9,*^

^†^ These authors contributed equally to this study.

^1^Department of Materials Science and Engineering, National Yang Ming Chiao Tung University, No. 1001, Daxue Rd., Hsinchu City 300093, Taiwan, ROC

^2^Department of Biomedical Engineering, National Yang Ming Chiao Tung University, No.155, Sec.2, Linong St., Taipei City 112304, Taiwan, ROC

^3^Department of Photonics, National Cheng Kung University, No.1, University Rd., Tainan City 701401, Taiwan, ROC

^4^Center of Applied Nanomedicine, National Cheng Kung University, No.1, University Rd., Tainan City 701401, Taiwan, ROC

^5^Department of Education, Taipei Veterans General Hospital, No.201, Sec. 2, Shipai Rd., Taipei City 11217, Taiwan, ROC

^6^Ph.D. Program in Medical Neuroscience, College of Medical Science and Technology, Taipei Medical University. 12F., Education & Research Building, Shuang-Ho Campus, No. 301, Yuantong Rd., New Taipei City 23564, Taiwan, ROC

^7^Department of Electronics and Electrical Engineering, National Yang Ming Chiao Tung University, No. 1001, Daxue Rd., Hsinchu City 300093, Taiwan, ROC

^8^Graduate Institute of Biomedical Science, China Medical University, No. 91, Xueshi Rd., Taichung City 404328, Taiwan, ROC

^9^ School of Dentistry, College of Dental Medicine, Kaohsiung Medical University, No. 100, Shiquan 1st Rd., Kaohsiung City 807378, Taiwan, ROC

*Correspondence should be addressed to either of the following:

Prof. Wei-Chen Huang

Department of Electronics and Electrical Engineering, National Yang Ming Chiao Tung University, Hsinchu City 300093, Taiwan, ROC

Email: [weichenh@nycu.edu.tw](mailto:weichenh@nycu.edu.tw)

Prof. You-Yin Chen

Department of Biomedical Engineering, National Yang Ming Chiao Tung University, Taipei City 112304, Taiwan, ROC

Email: [irradiance@so-net.net.tw](mailto:irradiance@so-net.net.tw) / [youyin.chen@nycu.edu.tw](mailto:youyin.chen@nycu.edu.tw)

Prof. San-Yuan Chen

Department of Materials Science and Engineering, National Yang Ming Chiao Tung University, Hsinchu City 300093, Taiwan, ROC

Email:

[sanyuanchen@nycu.edu.tw](mailto:sanyuanchen@nycu.edu.tw)

### **Note 1. High-magnification SEM image of the NT-PEI-ChR2 nanocomplex**


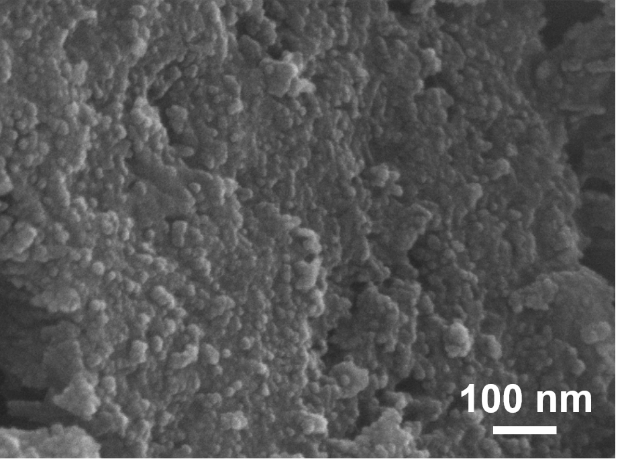


**Figure S1.** SEM image of the NT-PEI-ChR2 nanocomplex showing its surface morphology and nanoscale size distribution, further confirming successful complexation between NT-PEI and ChR2 plasmid DNA. **Note 2. Agarose gel retardation assay**

The binding efficiency of NT-PEI to ChR2 plasmid DNA was evaluated by an agarose gel electrophoresis assay. NT-PEI-ChR2 complexes were prepared at various *N/P* ratios (0.005k, 0.01k, 0.05k, 0.1k, and 1k), where the *N/P* ratio is defined as the molar ratio of amine nitrogen atoms in NT-PEI to phosphate groups in the ChR2 plasmid DNA. The samples were mixed and incubated at room temperature for 30 min to allow complete complexation. The complexes were then loaded onto a 1% (*w/v*) agarose gel containing GelRed nucleic acid stain and electrophoresed at 100 V for 30 min. DNA bands were visualized using a UV transilluminator to assess the degree of DNA migration retardation (**Figure S2**).


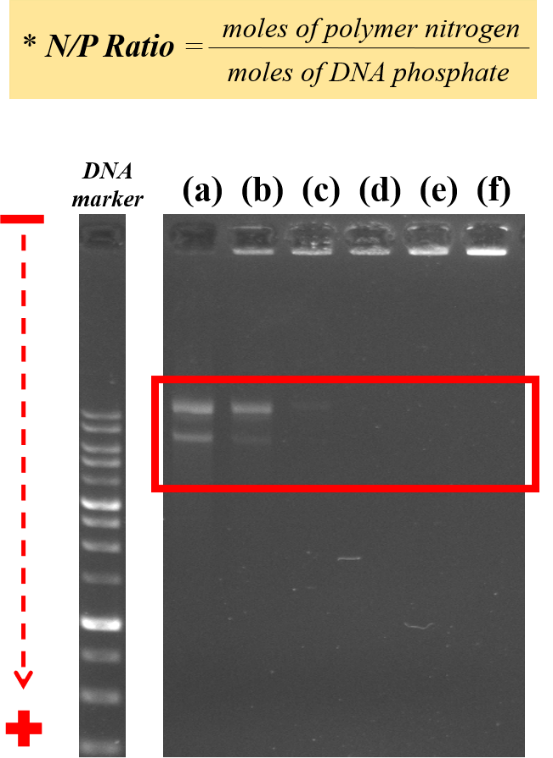


**Figure S2.** Agarose gel retardation assay demonstrating the binding efficiency of the NT-PEI-ChR2 nanocomplex at various *N/P* ratios. Lane (a): naked ChR2 plasmid DNA; lanes (b–f): NT-PEI-ChR2 complexes with increasing *N/P* ratios of 0.005k, 0.01k, 0.05k, 0.1k, and 1k, respectively. Complete retardation at an *N/P* ratio of 0.05k indicates full complexation and effective DNA binding by NT-PEI.

### **Note 3. *In vitro* cytotoxicity evaluation**

The PC-12 was cultured in Roswell Park Memorial Institute (RPMI) 1640 medium (Gibco, Thermo Fisher Scientific, MA, USA) supplemented with 10% fetal bovine serum (FBS), 100 µg/mL penicillin, and 100 µg/mL streptomycin. Cells were maintained at 37 °C in a humidified atmosphere containing 5% CO_2_.

For cytotoxicity evaluation, PC-12 cells were seeded in 96-well plates at a density of 1 × 10^4^ cells per well and allowed to adhere overnight. The culture medium was then replaced with fresh medium containing various concentrations (weight percentages: 0.01%, 0.05%, and 0.1%) of the NT-PEI-ChR2 complex. After 24 h of incubation with the gene vector, the medium was removed and cells were gently washed twice with PBS to eliminate residual complexes.

Subsequently, fresh RPMI medium containing the CCK-8 reagent (Cell Counting Kit-8, Abcam, Cambridge, UK) was added to each well and incubated for an additional 1 h at 37 °C. The absorbance was measured at 450 nm using a microplate spectrophotometer (Epoch^TM^ 2, BioTek Instruments Inc., Winooski, VT, USA). Cell viability was calculated relative to the untreated glass substrate control, which was defined as 100% viability.


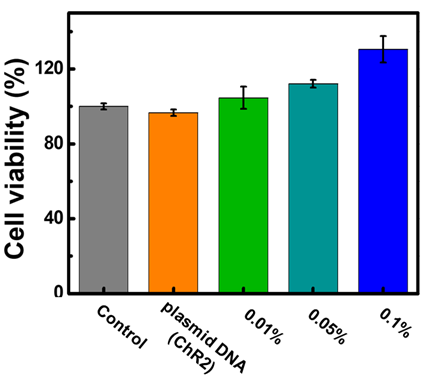


**Figure S3.** *In vitro* cytotoxicity assessment of the NT-PEI-ChR2 non-viral gene delivery complex in PC-12 cells, determined using the CCK-8 assay. The weight percentages indicate the ratio of NT-PEI-ChR2 (based on ChR2 vector weight) added to the culture medium. Cells were incubated with each formulation for 24 h. Data are presented as mean ± standard deviation (SD) (*n* = 3). The untreated glass substrate was used as the control to define 100% cell viability.

### **Note 4. EDX mapping analysis**

The elemental distribution of NT-PEI-ChR2 loaded within the 3D AuIO scaffold was characterized by cross-sectional scanning electron microscopy (SEM) combined with energy-dispersive X-ray (EDX) spectroscopy (JSM-7800F Prime, JEOL Ltd., Japan). Cross-sectional samples were prepared by fracturing the loaded AuIO films. EDX elemental maps for gold (***Au***), carbon (***C***), nitrogen (***N***), and phosphorus (***P***) were collected to confirm the presence and spatial distribution of the polymer carrier (NT-PEI) and ChR2 plasmid within the porous structure (**Figure S4**).


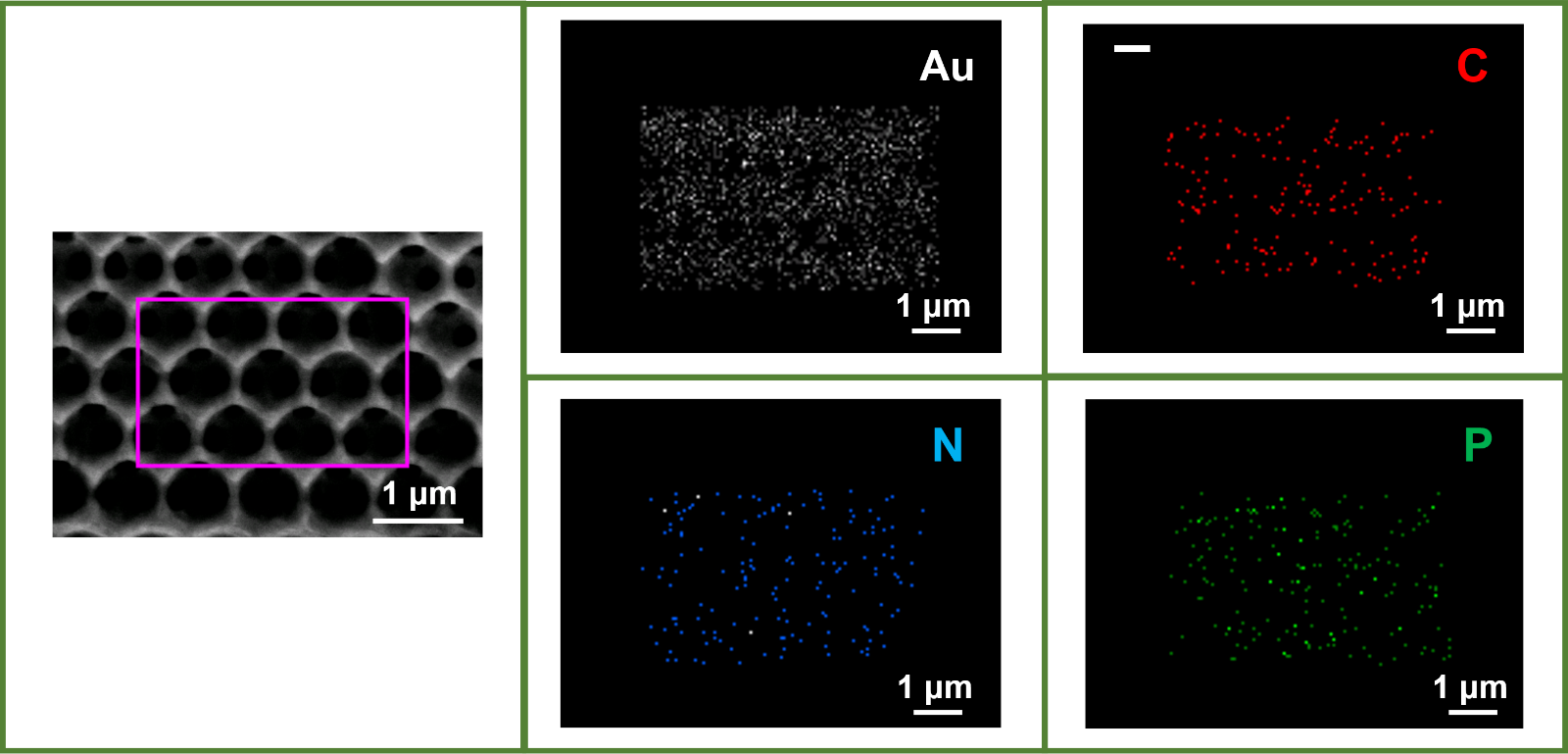


**Figure S4.** Cross-sectional SEM image and corresponding EDX elemental mapping of the NT-PEI-ChR2 nanocomplex loaded within the 3D AuIO structure. The SEM image (left) shows the highly porous architecture of the AuIO scaffold, while the EDX maps confirm the spatial distribution of key elements: Au (scaffold framework), ***C*** and ***N*** (from the NT-PEI polymer carrier), and ***P*** (from the ChR2 plasmid DNA). The co-localization of ***C***, ***N****,* and ***P*** within the porous matrix demonstrates successful encapsulation of the gene complex.

**Note 5. Fabrication of the planar Au nanofilm electrode**
 To fabricate the planar Au electrode, a gold nanofilm was electrodeposited onto ITO-coated glass substrates using a 5 mM HAuCl_4_ aqueous electrolyte. A constant electric field of 5 V/cm was applied for 10 min in a two-electrode configuration, with ITO (2 × 2 cm^2^) as the working electrode and a polished stainless-steel sheet (9 cm^2^) as the counter electrode.

Prior to deposition, the substrates were cleaned by sequential ultrasonication in ethanol and deionized water and dried under nitrogen flow to remove surface contaminants and promote uniform nucleation. The resulting gold layer exhibited a uniform nanogranular morphology with an estimated average grain size of ~80–100 nm, forming a dense and continuous coverage over the entire substrate.

Cross-SEM imaging (data not shown) revealed a deposited film thickness of approximately 450 nm, while top-view SEM (**Figure S5**) confirmed high nucleation density estimated at **~**2.3 × 10^10^ grains/cm^2^, with minimal lateral grain coalescence. The compact and non-porous nature of the film renders it ideal as a flat control to isolate the effects of surface topography on gene entrapment and release behaviors.


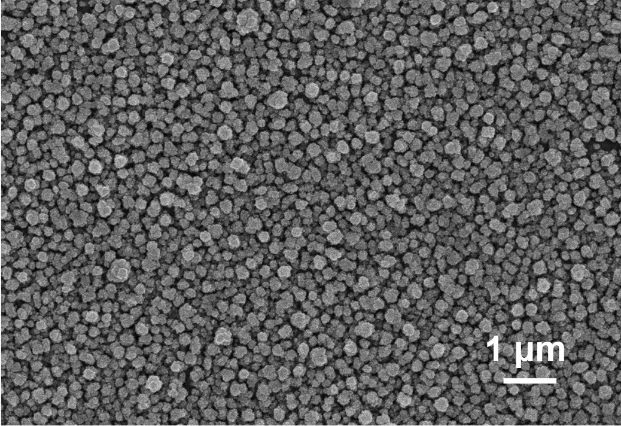


**Figure S5.** Top-view SEM image of the electrodeposited planar Au film on ITO substrate. The nanogranular structure shows a dense and continuous morphology with uniform grain distribution. The average grain diameter was estimated to be ~90 nm, and the nucleation density reached ~2.3 × 10^10^ grains/cm^2^. This structure serves as a flat control surface in comparative gene retention/release studies against 3D AuIO electrodes.

**Note 6. Release kinetics and mechanistic analysis based on the power law model**

The gene release kinetics were analyzed using the power law model:

$F=\frac{M_{t}}{M_{\infty}}\mathbb{=R}t^{\mathbb{N}}$ (**S1**)

where $F$ is the fractional release, defined as the ratio of the cumulative amount released at time $t$ ($M_{t}$​) to the total amount released at infinite time ($M_{\infty}$). The parameter $\mathbb{R}$ is a release rate constant characteristic of the system, and $\mathbb{N}$ is an exponent indicating the release mechanism and kinetics. By taking the natural logarithm of **Eq. (S1)** ^[1]^, the linearized form is obtained:

$\ln\left( \frac{M_{t}}{M_{\infty}} \right)=\ln\mathbb{R}+\mathbb{N}\ln t$ (**S2**)

The kinetic parameters $\mathbb{R}$and $\mathbb{N}$ determined from this model are summarized in **Table S1** and **Table S2**. In principle, the kinetic analysis was based on the initial 60% of the fractional release profiles ^[1-2]^. The Planar-E^+^ group was excluded due to significant fluctuations within the first min of its release profile. For the Planar-E^-^ group, as shown in **Table S1**, the calculated $\mathbb{N}$ value was 1.1, corresponding to *super case II transport*. This indicates that the release was predominantly governed by rapid macromolecular relaxation of the polymer chains ^[3]^. In contrast, the AuIO-E^-^ group exhibited an $\mathbb{N}$ value of 0.52, indicative of anomalous (non-Fickian) transport, suggesting that physical constraints within the 3D matrix limited both polymer swelling and chain relaxation ^[4]^. Consequently, the release behavior in the AuIO-E^-^ group reflects a nonlinear, time-dependent mechanism influenced by both diffusion/swelling and polymer relaxation within the filled 3D AuIO structure ^[3b, 4]^.

For the AuIO-E^+^ groups (including AuIO-50 μA, AuIO-100 μA, and AuIO-200 μA, as presented in **Table S2**), the $\mathbb{N}$ values were below 0.5, indicating Fickian diffusion-controlled release. This suggests that the rate of polymer chain relaxation was significantly faster than the rate of water permeation, resulting in a release process primarily governed by diffusion. Notably, as the stimulation current increased from 50 μA to 200 μA, the $\mathbb{N}$values decreased from 0.32 to 0.21 (**Table S2**), implying that higher current intensities enhanced polymer chain relaxation. This effect can be attributed to the continuous biphasic current driving redox transitions within the polymer, thereby modulating its swelling and dissolution behavior to facilitate gene release ^[5]^.

**Table S1.** Calculated kinetic constant ($\mathbb{R}$) and release exponent ($\mathbb{N}$) values obtained from fitting the power law equation to the initial 60% of the fractional release profiles shown in **Figure 2a**.

| Sample | kinetic constant $\mathbb{R}$ | release exponent $\mathbb{N}$ |
| --- | --- | --- |
| Planar-E^+^ | ~∞ | ~0 |
| Planar-E^-^ | 0.91 | 1.1 |
| AuIO-E^+^ | 2.72 | 0.27 |
| AuIO-E^-^ | 0.73 | 0.51 |

**Table S2.** Calculated kinetic constant ($\mathbb{R}$) and release exponent ($\mathbb{N}$) values obtained from fitting the power law equation to the initial 60% of the fractional release profiles shown in **Figure 2b**.

| Sample | kinetic constant $\mathbb{R}$ | release exponent $\mathbb{N}$ |
| --- | --- | --- |
| AuIO-200 μA | 3.22 | 0.21 |
| AuIO-100 μA | 2.76 | 0.27 |
| AuIO-50 μA | 2.33 | 0.32 |
| AuIO-E^-^ | 0.73 | 0.52 |

**Note 7. Electrochemical characterization of neural** **electrode array microelectrodes**

The capacitive and impedance properties of the neural electrode array microelectrodes, both with and without 3D AuIO surface modification, were comprehensively evaluated using a standard three-electrode configuration. All electrochemical measurements were performed using a CHI 614C electrochemical workstation (CH Instruments, Inc., Austin, TX, USA) in an electrolyte of 3 M NaCl to simulate a physiologically relevant ionic environment.

For each test, the microelectrode under investigation served as the working electrode. A high-purity platinum plate was employed as the counter electrode to ensure stable current flow without polarization effects. An Ag/AgCl electrode functioned as the reference electrode to provide a stable and well-defined reference potential throughout all measurements.

Cyclic voltammetry (CV) was conducted to assess the capacitive behavior. CV scans were performed over an appropriate potential window (−0.2 V to 0.6 V vs. Ag/AgCl) at 5 mV/sec to capture the charge storage characteristics and verify the pseudocapacitive contribution of the electrode modification. The resulting current–voltage curves were recorded and the enclosed area was analyzed to estimate the specific capacitance.

Electrochemical impedance spectroscopy (EIS) was carried out to investigate the frequency-dependent impedance characteristics. EIS measurements were performed by applying a small sinusoidal AC perturbation (typically 10 mV RMS) over a frequency range spanning from 0.1 Hz to 10 kHz. The impedance spectra were collected at open-circuit potential and fitted using an equivalent circuit model to extract parameters such as solution resistance, charge transfer resistance, and double-layer capacitance.

All tests were repeated at least three times for each electrode type to ensure reproducibility. The acquired data were processed and plotted using the built-in analysis tools provided by the CHI 614C software suite, and representative CV curves and impedance spectra are presented in **Figure S6**.


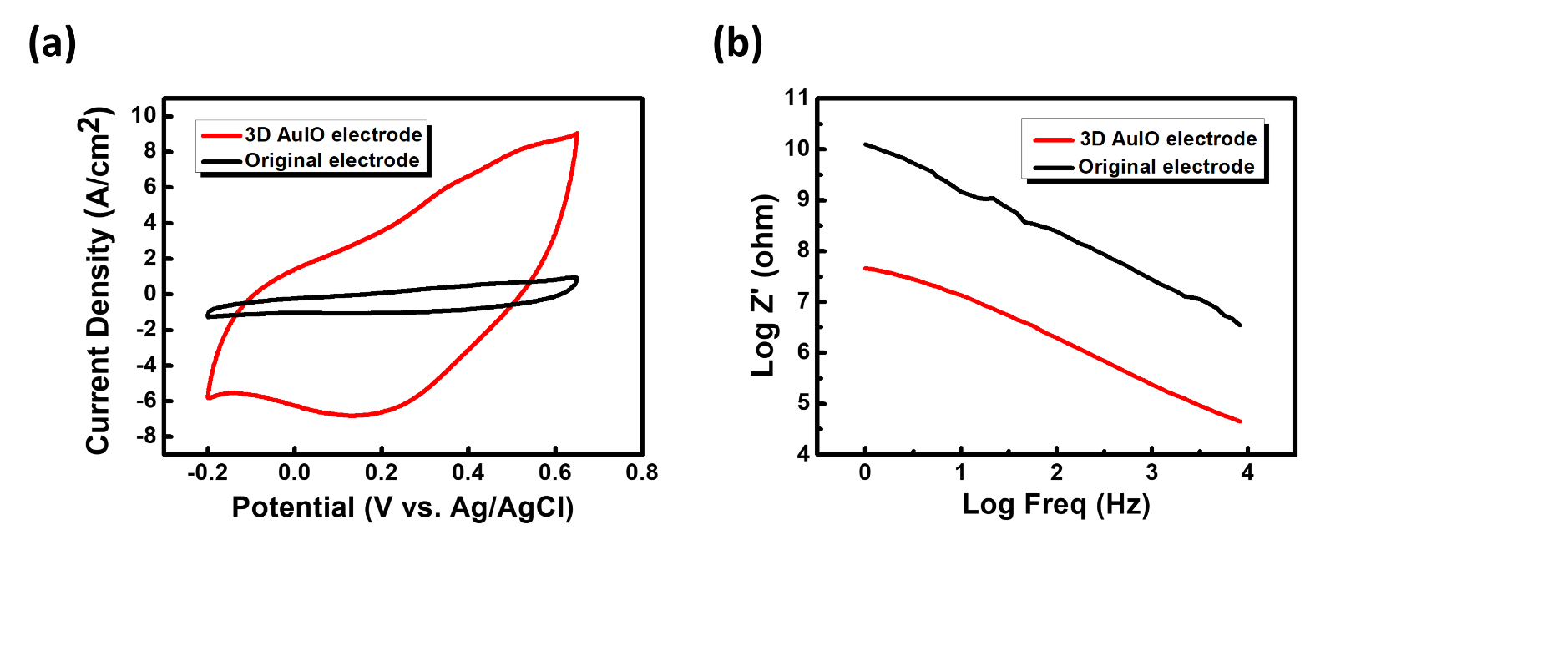


**Figure S6.** **Electrochemical performance comparison of neural electrode array microelectrodes with and without 3D AuIO surface modification**. (a) CV curves acquired in 3 M NaCl solution using a three-electrode setup, showing enhanced capacitive behavior for the 3D AuIO-modified microelectrode (red curve) compared to the unmodified original electrode (black curve). (b) EIS spectra indicating reduced impedance magnitude across a wide frequency range for the 3D AuIO-modified microelectrode, demonstrating improved charge transfer and interfacial properties.

**Note 8. Flow cytometry for transfection efficiency quantification**
 Supplementary flow cytometry analysis further confirmed the trend observed in fluorescence microscopy and **Figure 2c**. As shown in **Figure S7**, the fluorescence intensity distribution of mCherry-positive cells clearly shifted rightward in the NT-PEI-ChR2-E^+^ group compared to all other groups, indicating the highest gene expression level. The PEI-ChR2-E^+^ group also demonstrated a rightward shift relative to its non-electroporated counterpart (PEI-ChR2-E^-^), confirming the role of electrical stimulation in enhancing transfection. Notably, groups without the NT modification (i.e., PEI-ChR2 or ChR2 alone) exhibited weaker shifts and lower peak counts, while the NT-PEI-ChR2-E^-^ group showed moderate improvement over conventional PEI-ChR2 due to NT-mediated targeting alone. Control and ChR2-E^-^ groups exhibited minimal signal. Collectively, these flow cytometry profiles quantitatively validate the superior transfection performance of the NT-PEI-ChR2-E^+^ strategy, driven by both enhanced cellular targeting and electroporation.


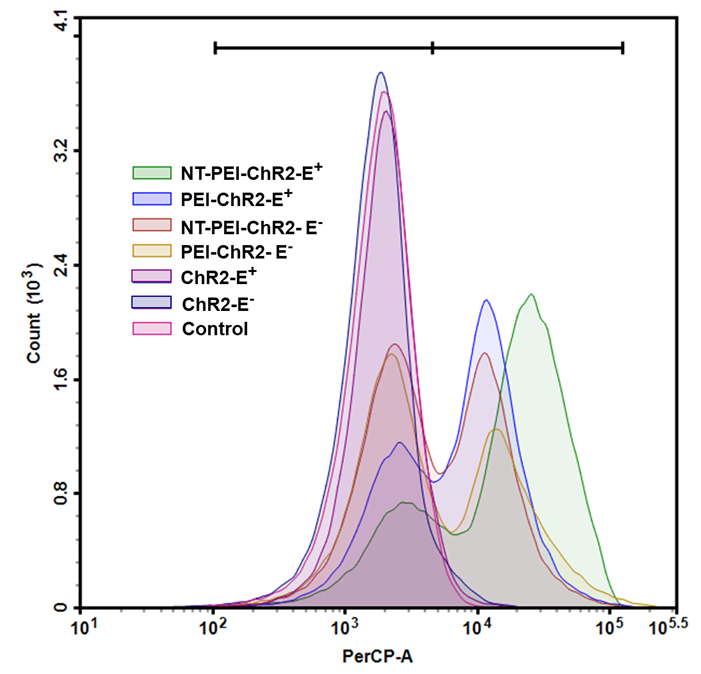


**Figure S7.** Flow cytometry analysis of transfection efficiency for mCherry-labeled ChR2 plasmids under different delivery conditions. Groups include: NT-PEI-ChR2-E^+^, PEI-ChR2-E^+^, NT-PEI-ChR2-E^-^, PEI-ChR2-E^-^, ChR2-E^+^, ChR2-E^-^, and untreated control. The rightward fluorescence intensity shift indicates increased intracellular mCherry expression. The NT-PEI-ChR2-E^+^ group exhibited the highest fluorescence signal, confirming enhanced delivery efficiency through combined NT-targeting and localized electroporation.

**Note 9. Adhesion stability of UCNP coatings under simulated brain insertion conditions using GelMA hydrogel encapsulation**


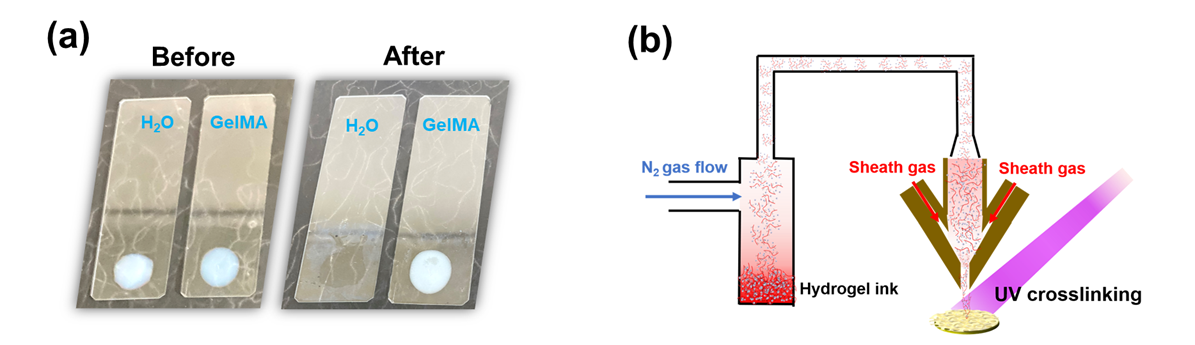


**Figure S8.** (a) Comparison of coating retention between water-based UCNP solution and UV-crosslinked GelMA-based UCNP hydrogel after insertion into a 1 wt% agarose brain phantom. The GelMA-based coating remains intact post-insertion, while the water-based UCNPs are visibly removed. (b) Schematic illustration of the coaxial gas-sheath-assisted hydrogel printing and UV-crosslinking process for forming a stable UCNP-GelMA coating on microelectrodes.

**Note 10. Plasmon-enhanced upconversion luminescence of UCNPs on 3D AuIO nanostructures for low-power optogenetic stimulation**


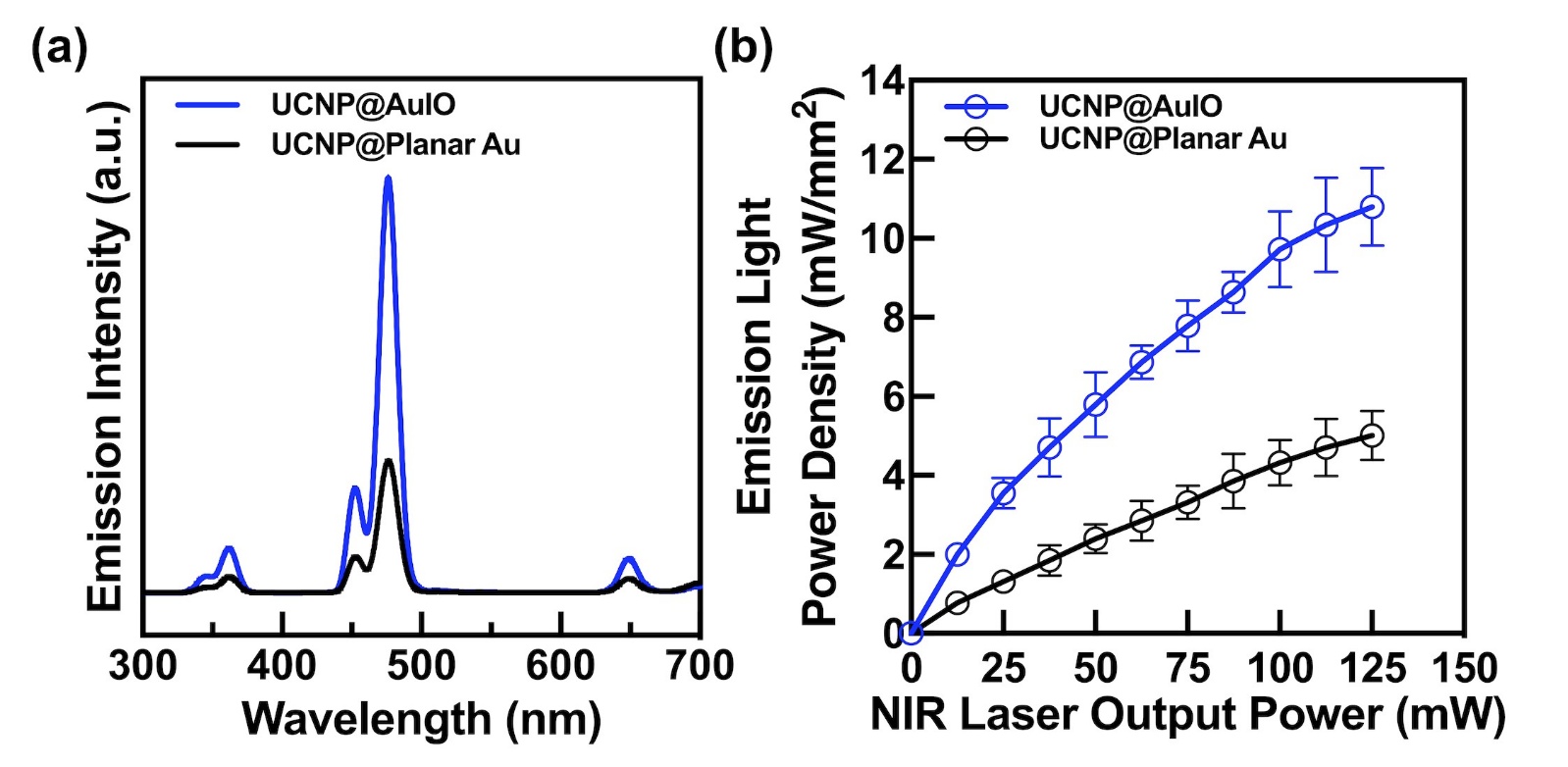


**Figure S9**. (a) Photoluminescence spectra comparing the UCL emission from UCNP@AuIO-modified and UCNP@Planar Au nanofilm electrodes of the ITO substrate. The UCNP@AuIO exhibited a peak intensity of approximately 450 a.u. at ~475 nm, with an estimated integrated intensity of ~8500 a.u. (450–500 nm). In contrast, the UCNP@Planar Au electrode showed a peak of ~230 a.u. and an integrated intensity of ~4200 a.u., corresponding to a ~2-fold enhancement in blue output from the UCNP@AuIO electrode. This enhancement is attributed to LSPR amplification induced by the 3D AuIO nanostructure. (b) Measured blue emission power at 475 nm as a function of increasing NIR laser output power. Under 50 mW excitation, the UCNP@AuIO electrode achieved a blue-light power density of ~5.86 mW/mm^2^. The UCNP@Planar Au electrode generated a power density of ~2.47 mW/mm^2^. The UCNP@AuIO device thus reaches optogenetically relevant emission levels at lower input powers, demonstrating its potential for safe, effective, and wireless optogenetic stimulation.

#### **Note 11. Aerosol jet printing of UCNP coating and gene nanocomplex onto 3D AuIO-modified microelectrodes**

Aerosol jet printing was employed for precise, maskless deposition of both the GelMA-based UCNP hydrogel (**Figure S10**) and PEI–NT–ChR2-mCherry nanocomplex onto the 3D AuIO-modified microelectrodes of the neural electrode array. All printing was performed using a commercial **Optomec Aerosol Jet HD2 system** (Optomec Inc., Albuquerque, NM, USA), equipped with an ultrasonic atomizer and pneumatic aerosol delivery module.

##### **Table S3. SGelMA-UCNP deposition parameters (optical transducer layer)**

| Parameter | Specification |
| --- | --- |
| Ink formulation | GelMA (3 wt%) + UCNPs (2 mg/mL, NaYF_4_:Yb^3+^, Tm^3+^, ~30 nm) + Irgacure 2959 (0.5 wt%) |
| Nozzle size | 150 μm |
| Sheath gas (N_2_) flow rate | 35 sccm |
| Carrier gas flow rate | 25 sccm |
| Print speed | 2 mm/sec |
| Platen (substrate) temperature | 30 °C |
| Post-deposition UV crosslinking | 365 nm UV, 10 mW/cm^2^, for 120 sec |
| Resolution | Down to 10 μm trace width, 20 μm spacing |
| Standoff distance (print gap) | Up to 5 mm (enables conformal printing on 3D AuIO) |
| Motion resolution | 0.1 μm |

The UCNP-GelMA formulation was atomized and delivered *via* coaxial gas flow, allowing high-resolution (∼20 μm linewidth) patterning directly on Channels #2 and #3. The resulting coatings exhibited excellent conformality and adhesion on the porous AuIO structure.

##### **Table S4. PEI–NT–ChR2-mCherry nanocomplex deposition (gene vector layer)**

| Parameter | Specification |
| --- | --- |
| Ink formulation | Aqueous NT-PEI-ChR2 nanocomplex (ChR2 plasmid: 2 μg/mL; *N/P* = 0.05k) |
| Nozzle size | 150 μm |
| Sheath gas (N_2_) flow rate | 35 sccm |
| Carrier gas flow rate | 20 sccm |
| Print speed | 1.5 mm/sec |
| Platen (substrate) temperature | Ambient (RT, ~25 °C) |
| Deposition area | Targeted microelectrodes (e.g., Ch #1 and Ch #4 of neural electrode array) |
| Drying condition | Air-dried for 10 min in sterile environment |
| Resolution (machine capability) | Down to 10 μm trace width, 20 μm spacing |
| Standoff distance (print gap) | Up to 5 mm (suitable for AuIO 3D surface) |
| Motion resolution | 0.1 μm |

Aerosol jet printing of the nanocomplex was carried out selectively on Channels #1 and #4. The non-contact nature of the aerosol jet printing ensured that the delicate nanostructure of the 3D AuIO microelectrodes remained intact. After deposition, the patterns were air-dried under sterile conditions for 10 min to ensure nanocomplex immobilization prior to surgical implantation.

The overall patterning resolution was maintained within **± 0.1 μm positional accuracy**, with minimal overspray due to optimized sheath-to-carrier gas ratios. Real-time optical alignment was performed using the integrated vision system on the aerosol jet printing platform to ensure precise electrode targeting.


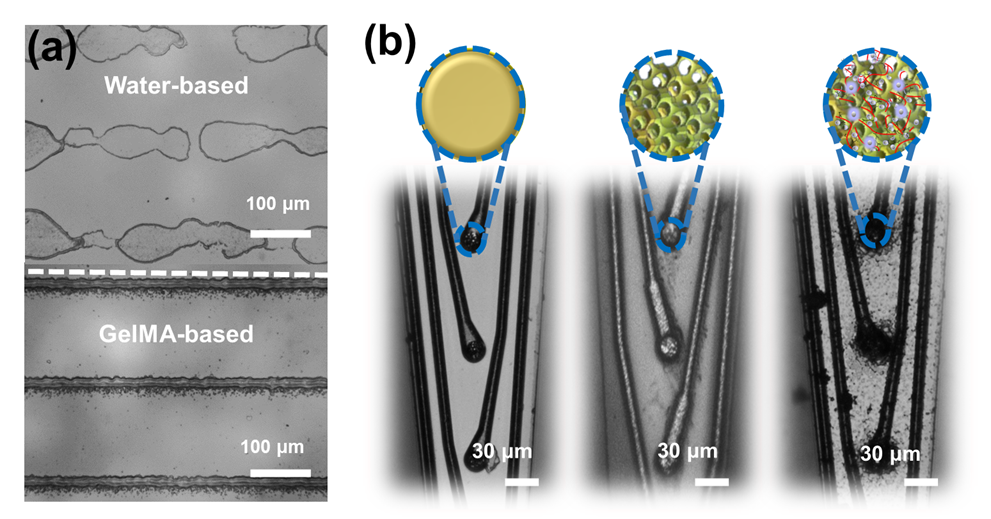


**Figure S10. Evaluation of UCNP coating stability and structural integration on 3D AuIO-modified microelectrodes of neural electrode array.** (a) Optical microscopy images showing the morphology of patterned UCNP arrays printed using water-based ink (top) and GelMA-based hydrogel ink (bottom) by aerosol jet printing. The water-based UCNP coating exhibits discontinuous and unstable deposition, whereas the GelMA-based UCNP arrays form uniform, linear features with widths of approximately 18 μm, demonstrating enhanced patterning fidelity and adhesion. (b) Optical images of neural probes sequentially modified with 3D AuIO nanostructures and coated *via* aerosol jet printing with GelMA-embedded UCNPs. The three neural electrode array represent different coating stages: (left) bare planar microelectrode, (middle) 3D AuIO-modified microelectrode, and (right) 3D AuIO microelectrode coated with GelMA-UCNP hydrogel. Insets illustrate the structural evolution from flat surface to porous scaffold and finally to nanocomposite integration, confirming the mechanical stability and surface conformity of the UCNP hydrogel on the 3D microelectrode architecture.

**Note 12. Schematic and photographic setup for quantitative UCL power measurements using Micro-Raman spectrometer**


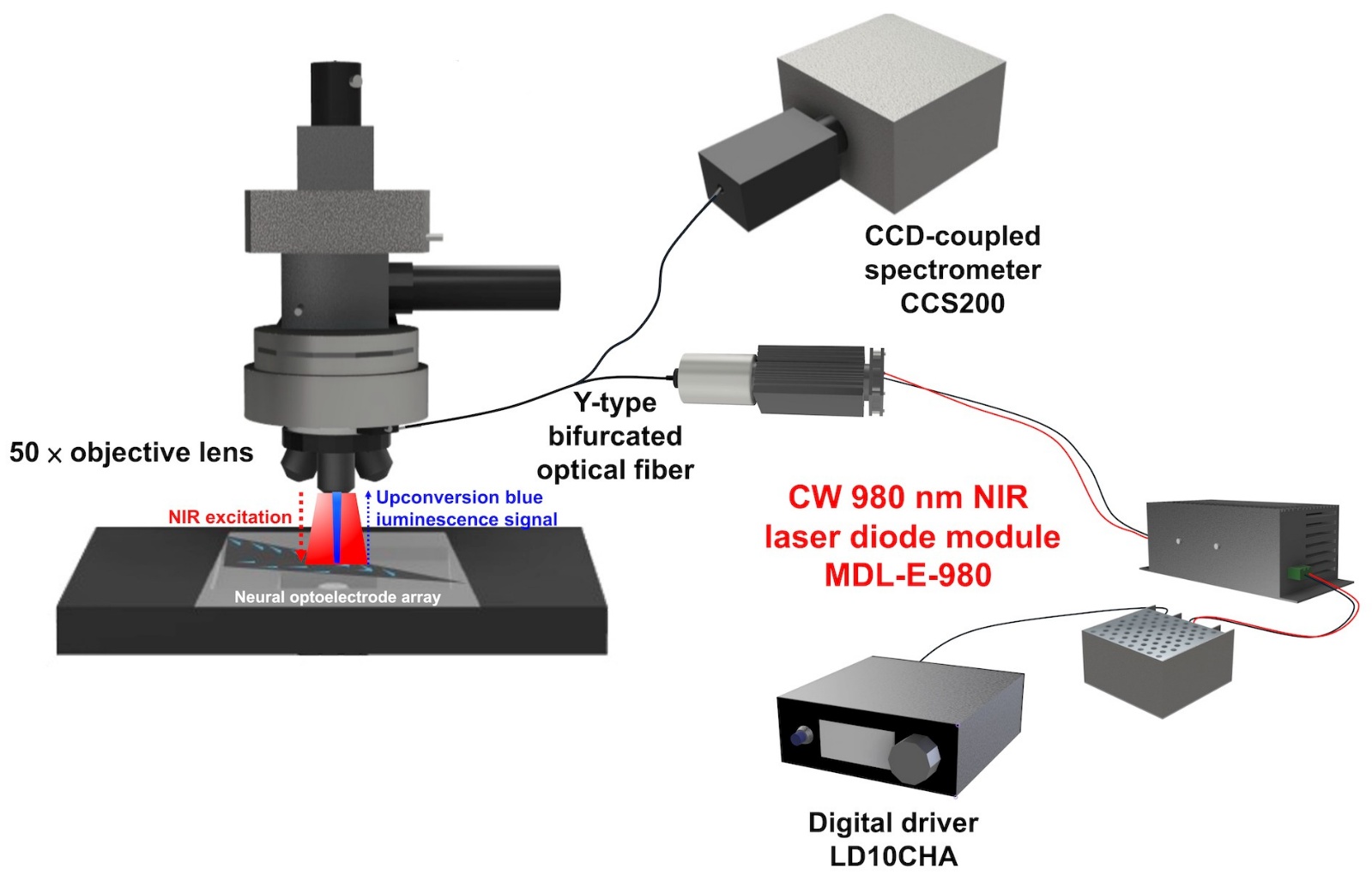


**Figure S11**. Schematic of the integrated optical system featuring a Y-type bifurcated optical fiber coupled with a 50× objective lens, enabling simultaneous delivery of 980 nm NIR excitation and collection of upconversion blue luminescence signals. This setup allows for *in situ* optical measurement of UCNP@3D AuIO, UCNP@Au, and bare Au microelectrodes within the neural electrode array. One branch of the bifurcated fiber is connected to the NIR laser diode module (Model: MDL-E-980, New Industries Optoelectronics Tech. Co., Ltd., Changchun, China), while the other branch is linked to a CCD-coupled spectrometer (Model: CCS200, Thorlabs Inc., Newton, NJ, USA) for spectral acquisition. The laser output is regulated via a digital driver (Model: LD10CHA, New Industries Optoelectronics Tech. Co., Ltd.), and the optical power (400–1100 nm) emitted from the sample is measured using a fiber-coupled photodiode sensor (Model: S151C) connected to a power meter (Model: PM100D, Thorlabs Inc., Newton, NJ, USA). This configuration enables precise, spatially resolved quantification of UCNP luminescence at individual microelectrode sites, with or without 3D AuIO nanostructure modifications.

**Note 13. Technical comparison: our work *vs.* other works**

**Table S5. Technical comparison between our UCNP@3D AuIO platform and prior remote neuromodulation systems**

| Comparison Aspect | Our Work (UCNP@3D AuIO) | Chen et al., 2018^[6]^ | Lin et al., 2017^[7]^ | Liu et al., 2021^[8]^ | Jin et al., 2025^[9]^ |
| --- | --- | --- | --- | --- | --- |
| Gene Delivery | Non-viral NT-PEI-ChR2 (efficient & safe) | No gene delivery | No gene delivery | No gene delivery | No gene delivery (relies on photogenerated current) |
| Stimulation Mechanism | UCNP@GelMA + 3D AuIO with LSPR-enhanced NIR-to-blue conversion | Free UCNP nanogels (external NIR activation) | Injectable UCNP with LiYF_4_ matrix (internal heating) | Lanthanide-doped particles with photothermal effect | UCNP + WO_3-x_ hybrid for photocurrent generation |
| Functional Integration | Fully integrated: gene delivery + stimulation + electrophysiology | UCNP delivery only | Photothermal stimulation only | Stimulation only, no sensing | Stimulation only, no feedback |
| Efficiency & Depth | 30 mW/mm^2^ NIR → ~1.5 mm DG activation via LSPR upconversion | Limited (~400 μm cortical) | Moderate (~1.0 mm), particle-distribution dependent | Shallow (surface-focused) | Moderate (~1.2 mm), low upconversion yield |
| Closed-loop Capability | Yes, with real-time electrophysiological recording | No | No | No | No |
| Target Specificity | Opsin-based, neuron-specific via NT-PEI transfection | None | None | None | None |
| Material Integration | Porous AuIO + GelMA-stabilized UCNPs, implantable and biocompatible | UCNPs in nanogel form | Free-floating UCNP clusters | Dispersed particles | Mixed particle coatings, less precise |

**Note 14. Comparative thermal imaging of conventional micro-LED and UCNP@AuIO-based electrode array**

**The micro-LED probe, based on the Cree TR2432™ chip (470 nm emission, >24 mW radiant flux at 20 mA), was observed to induce localized temperature elevation during continuous blue light operation (Figure S14a), consistent with previous findings that even low-power blue illumination (~1.2 mW/mm^2^) can increase brain temperature by ~2 °C ^[10]^.**

**In contrast, our system employs spatially confined upconversion at the electrode interface, where externally applied 980 nm NIR light is converted locally into visible emission Figure S14b. Under a burst-mode, low-duty-cycle stimulation protocol (125 ms pulses at 4 Hz, 12% duty), with a power density of 30 mW/mm^2^ on brain surface, thermal imaging revealed no significant temperature rise at the probe-tissue interface. These results align with prior reports ^[11]^ indicating that appropriately modulated NIR light produces only mild (<1 °C) and biologically safe heating effects *in vivo*.**


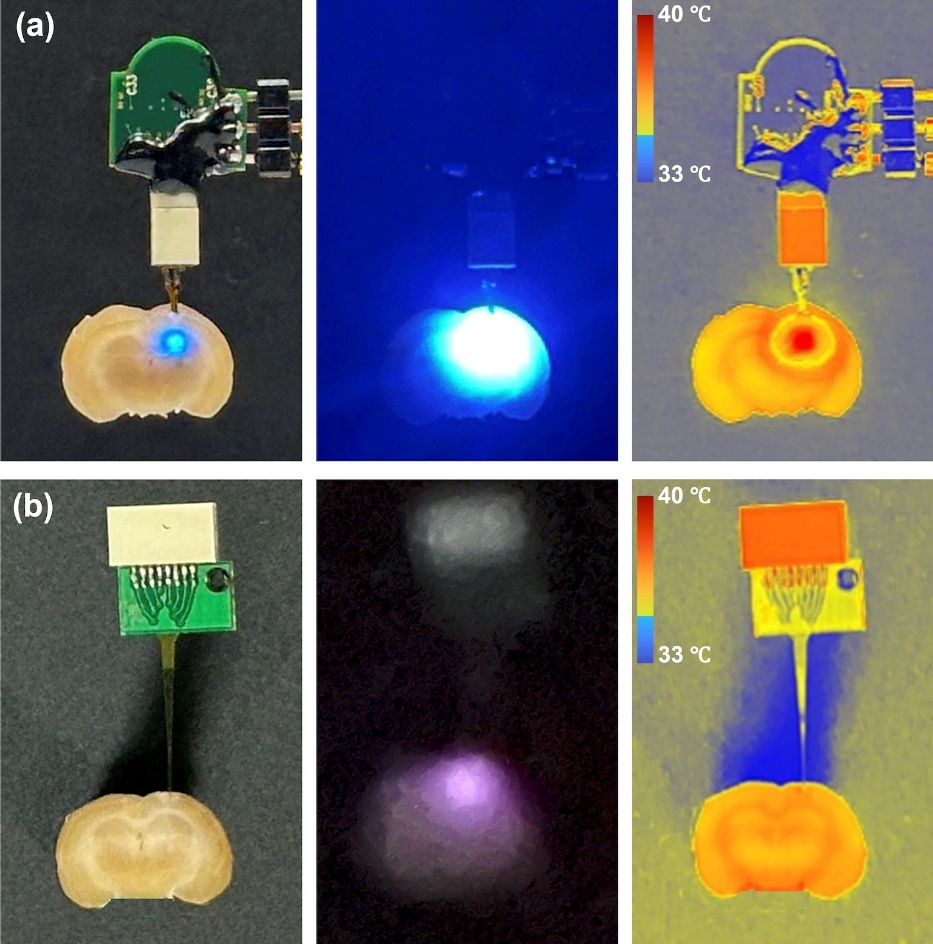


**Figure S14**. **Comparative optical, luminescence, and thermal imaging of a conventional micro-LED neural probe and the UCNP@AuIO-modified electrode array**. (a, left) Photograph of a conventional micro-LED neural probe inserted into brain tissue. (a, center) Optical image showing blue light emission (~470 nm) from the micro-LED during operation. (a, right) Infrared thermographic image recorded during pulsed blue light emission (4 Hz). A distinct localized temperature elevation is observed at the emission site, consistent with light-induced photothermal heating in tissue. (b, left) Photograph of the UCNP@AuIO-modified electrode array placed on brain tissue. (b, center) NIR-triggered upconversion luminescence image acquired during burst-mode stimulation (125 ms pulse width, 4 Hz frequency, 12% duty cycle), under a brain surface-applied 980 nm laser beam (power density: 30 mW/mm^2^). Blue emission is generated locally at the electrode tip. (b, right) Thermal image captured during the same stimulation protocol. No measurable temperature increase is observed at the UCNP@AuIO electrode array tip, indicating negligible local heating under the applied near-infrared stimulation conditions.

**Note 15. Degradation behavior and mechanical stability of the GelMA–UCNP printable coating**

To evaluate the structural integrity and degradation behavior of the UV-crosslinked GelMA–UCNP printable ink coating, a thin hydrogel film was prepared on an ITO glass substrate by replicating the jet-printing deposition sequence. Specifically, the GelMA-based hydrogel precursor was dispensed onto the substrate, followed by removal of excess liquid using a nitrogen gas stream, and immediate photo-crosslinking under UV light (10 mW cm^-2^ for 120 sec, see **Table S3**).

The coated samples were incubated at 37 °C in a brain phantom (1% Agarose) for 14 days. At 2-day intervals, the samples were removed, briefly blotted to remove surface water, and weighed to determine the wet mass. Simultaneously, digital photographs were taken to monitor the morphological changes in the film.

As shown in **Figure S15a**, the hydrogel film remained macroscopically intact and well-adhered to the ITO substrate throughout the first week. No visible delamination, peeling, or fragmentation was observed. The film exhibited a transparent and uniform appearance during this period. By day 14, signs of surface roughening and partial erosion became apparent, indicating the onset of hydrolytic degradation.

The quantitative mass retention data (**Figure S15b**) show an initial minor increase in mass due to water uptake and swelling, reaching a plateau (~5-8% swelling) within the first 6 days. After day 12, the wet mass gradually declined, reflecting the progressive degradation of the polymer network. These results are consistent with the behavior of surface-attached hydrogel films, which are known to swell less and degrade more slowly than bulk hydrogels due to limited solvent diffusion and constrained chain mobility.

To further assess the mechanical robustness of the printed coating, a repeated insertion–removal test was performed using hydrogel-coated substrates interfaced with a standard contact setup. Over multiple insertion/removal cycles, no delamination or damage was observed on the hydrogel layer, as confirmed by photographic inspection (**Figure S15**). The structural adhesion and conformal contact of the coating remained intact, highlighting the coating’s high resistance to interfacial shear forces, exhibiting excellent long-term structural and mechanical stability under physiological conditions.


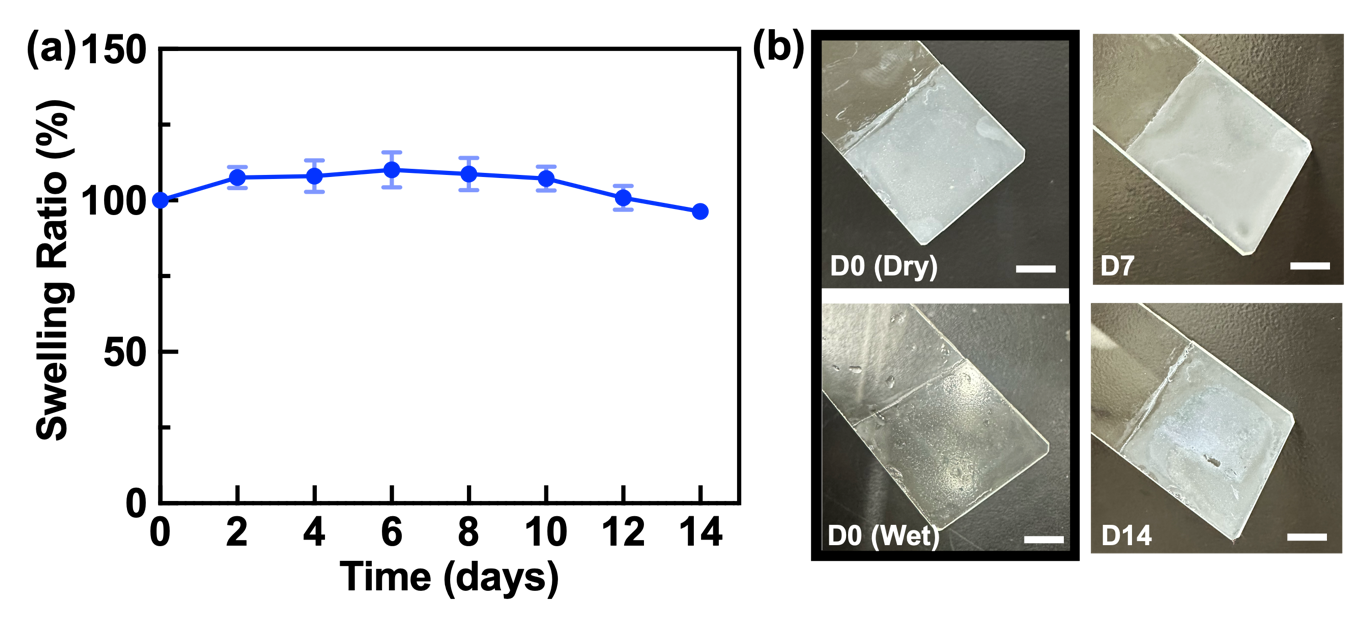


**Figure S15**. (a) Swelling Ratio as a function of immersion time, showing minor swelling over the first week, followed by gradual mass loss, consistent with slow hydrolytic degradation. (b) Representative degradation images of the GelMA printable ink coating on ITO substrate during a 14-day incubation in a brain phantom (37 °C). The hydrogel layer remains intact over a week, with visible degradation initiating at day 14. (All scale bar presents a scale of 1 cm)

**References**:

[1] P. L. Ritger, N. A. Peppas, *Journal of controlled release* **1987**, 5, 23.

[2] R. W. Korsmeyer, R. Gurny, E. Doelker, P. Buri, N. A. Peppas, *International Journal of Pharmaceutics* **1983**, 15, 25.

[3] a) P. Colombo, R. Bettini, N. A. Peppas, *J Control Release* **1999**, 61, 83; b) J. Siepmann, N. A. Peppas, *Int J Pharm* **2011**, 418, 6.

[4] S. Dash, P. N. Murthy, L. Nath, P. Chowdhury, *Acta Pol Pharm* **2010**, 67, 217.

[5] a) J. K. Patra, G. Das, L. F. Fraceto, E. V. R. Campos, M. d. P. Rodriguez-Torres, L. S. Acosta-Torres, L. A. Diaz-Torres, R. Grillo, M. K. Swamy, S. Sharma, S. Habtemariam, H.-S. Shin, *Journal of Nanobiotechnology* **2018**, 16, 71; b) C. L. Weaver, J. M. LaRosa, X. Luo, X. T. Cui, *ACS Nano* **2014**, 8, 1834.

[6] S. Chen, A. Z. Weitemier, X. Zeng, L. He, X. Wang, Y. Tao, A. J. Huang, Y. Hashimotodani, M. Kano, H. Iwasaki, *Science* **2018**, 359, 679.

[7] X. Lin, Y. Wang, X. Chen, R. Yang, Z. Wang, J. Feng, H. Wang, K. W. C. Lai, J. He, F. Wang, P. Shi, *Adv Healthc Mater* **2017**, 6.

[8] X. Liu, H. Chen, Y. Wang, Y. Si, H. Zhang, X. Li, Z. Zhang, B. Yan, S. Jiang, F. Wang, *Nature communications* **2021**, 12, 5662.

[9] S. Jin, J. Li, L. Jiang, Y. Ye, C. Ma, Y. Yang, H. Su, L. Gao, M. Ni, Y. Zhao, Y. Tian, G. Li, J. Shi, K. Zhang, P. Tang, Y. Yuan, B. Lai, M. Chen, L. Sun, *Sci Adv* **2025**, 11, eadt4771.

[10] S. B. Goncalves, J. M. Palha, H. C. Fernandes, M. R. Souto, S. Pimenta, T. Dong, Z. Yang, J. F. Ribeiro, J. H. Correia, *Micromachines (Basel)* **2018**, 9.

[11] S. Chen, A. Z. Weitemier, X. Zeng, L. He, X. Wang, Y. Tao, A. J. Y. Huang, Y. Hashimotodani, M. Kano, H. Iwasaki, L. K. Parajuli, S. Okabe, D. B. L. Teh, A. H. All, I. Tsutsui-Kimura, K. F. Tanaka, X. Liu, T. J. McHugh, *Science* **2018**, 359, 679.
